# Genome-Wide Association Analysis of Adaptation Using Environmentally Predicted Traits

**DOI:** 10.1371/journal.pgen.1005594

**Published:** 2015-10-23

**Authors:** Joost van Heerwaarden, Martijn van Zanten, Willem Kruijer

**Affiliations:** 1 Biometris, Wageningen University, Wageningen, The Netherlands; 2 Plant Production Systems, Wageningen University, Wageningen, The Netherlands; 3 Molecular Plant Physiology, Institute of Environmental Biology, Utrecht University, Utrecht, The Netherlands; Georgia Institute of Technology, UNITED STATES

## Abstract

Current methods for studying the genetic basis of adaptation evaluate genetic associations with ecologically relevant traits or single environmental variables, under the implicit assumption that natural selection imposes correlations between phenotypes, environments and genotypes. In practice, observed trait and environmental data are manifestations of unknown selective forces and are only indirectly associated with adaptive genetic variation. In theory, improved estimation of these forces could enable more powerful detection of loci under selection. Here we present an approach in which we approximate adaptive variation by modeling phenotypes as a function of the environment and using the predicted trait in multivariate and univariate genome-wide association analysis (GWAS). Based on computer simulations and published flowering time data from the model plant *Arabidopsis thaliana*, we find that environmentally predicted traits lead to higher recovery of functional loci in multivariate GWAS and are more strongly correlated to allele frequencies at adaptive loci than individual environmental variables. Our results provide an example of the use of environmental data to obtain independent and meaningful information on adaptive genetic variation.

## Author Summary

Finding genes involved in adaptation to the environment has long been of interest to evolutionary biologists and ecologists. Most commonly, researchers look for loci whose differences in allelic state correlate with differences in a particular trait or environmental variable such as temperature. The implicit assumption behind such methods is that natural selection by the environment will shape variation in adaptive traits through associated changes in allele frequencies. This means that both environmental and phenotypic variation are relevant for detecting adaptive genes, although we have incomplete knowledge of how the two types of variation relate to adaptation. Here we present a method that aims to identify adaptive genes by combining phenotypic and environmental data. We first predict trait variation from a set of environmental variables as a way to extract the most biologically relevant information from the environment and then look for genes associated with both the predicted and observed trait. Using simulations and published data from the model plant *Arabidopsis thaliana*, we show that this approach may find adaptive genes more effectively compared to existing methods. We also demonstrate that predicted traits can be used to identify relevant loci in individuals for which no phenotypic data is available.

## Introduction

The genetic basis of environmental adaptation in natural and agricultural populations is a topic of growing interest and urgency. Conventionally, the search for adaptive genes involves testing for associations of genomic markers with either ecologically relevant traits measured in common garden experiments [1] [2] [3] [4] or with environmental variables [5] [6] [7] [4] [8]. These two approaches reflect the assumption that traits, environment and genotype are correlated due to natural selection, as is indeed expected under local adaptation [9] [10] [11]. In practice, observations and measurements are subject to error and may not accurately reflect the actual variables involved in adaptation [6]. At best therefore, empirical data on traits and environment provide independent approximations of the parameters defining ecological adaptation, offering limited power to detect causative genes when used in isolation. An obvious improvement would be to combine both types of data to better approximate the adaptive process. One example is to identify the most probable selective forces from a set of environmental variables based on their correlation with traits of interest and use these variables in association mapping, as was done recently in *Arabidopsis thaliana* [7]. Although attractive, the reliance on single variables means that this method cannot account for more complex relations between traits and the environment and makes limited use of the independent information provided by trait and environmental data.

An alternative approach, which we explore here, is to extract information from ecological data by modeling traits as a function of multiple environmental variables [12] [13] and to use the resulting trait prediction, conjointly with the observed trait, in a bivariate analysis of genetic association. The reasoning behind this idea is as follows. We start from the usual assumption that individuals from different geographic locations express location-specific, genetically determined trait values that are optimal with respect to some combination of environmental conditions in their native habitat. Furthermore, as in other studies on environmental association, we assume that clinal variation in selective forces causes corresponding differences in gene frequencies across the landscape. Under these assumptions, the value of a trait and its defining selective environment can be treated as two correlated aspects of an individual’s phenotype with a shared genetic basis.

In the same way, observed variation in an adaptive trait and a function of environmental variables explaining part of this variation can be treated as two genetically correlated characteristics that are effectively repeated measurements of the underlying selective environment. As has been shown for other genetically correlated traits, such repeated measurements may be combined to increase the power to detect common causative loci by testing for genetic associations with both traits simultaneously using a multi-trait mixed model (MTMM) [14] [15]. We propose that testing for genetic loci with an effect on both observed and predicted traits provides more power to detect genes of adaptive significance than mapping on individual traits or environmental variables separately. In addition, environmentally predicted traits may be used in univariate association mapping to map adaptive loci in individuals for which only environmental data is available. We will refer to these two applications of predicted traits as bivariate- and univariate Environmentally predicted Trait Mapping (ETM) throughout the paper. We demonstrate the potential of bivariate ETM by computer simulations and evaluate its performance using phenotypic and high-density SNP data from a published association study on flowering time in *Arabidopsis thaliana* [1]. Flowering time is known to affect fitness in *A. thaliana* [16] and shows strong geographic variation [17], making it an ideal trait for our purposes. Moreover, its genetic and molecular basis is well understood [18] [19]. We compare the power of bivariate ETM to recover known flowering genes to that of conventional univariate association methods using single traits or environmental variables. In addition, we use univariate ETM to map flowering genes in individuals without available phenotypic data [7], an approach that may offer potential for allele mining germplasm collections for adaptive variation.

## Results

### Environmental Trait Mapping (ETM)

ETM first models the observed phenotype as a function of environmental data, producing a combination of the environmental variables which we call the predicted phenotype. The trait prediction model is fit on the set of accessions for which both phenotypic and environmental data are available, but the resulting prediction can be extended to the accessions for which there are only environmental data. In case of non-constant prediction, bivariate ETM then performs multitrait association mapping on the observed and predicted phenotype, using all available accessions. In univariate ETM we perform single trait association mapping for the accessions with missing phenotypic data.

### Simulations

As proof of concept, we simulated a simple scenario in which an adaptive trait is modeled as a linear function of a random subset of ten out of 30 simulated environmental variables (Materials and Methods). The frequency of the causative SNP was set to be a monotone function of the true adaptive trait. The observed trait was then defined as the sum of a SNP effect and polygenic and residual noise.

Four trait prediction methods were implemented: linear model (LM) prediction with backward variable selection, elastic nets (EN) [20], random forests (RF) [21] and canonical correlation analysis (CCA) [22]. For comparison, we also performed bivariate analysis using the trait and the most correlated environmental variable, as well as univariate GWAS on the trait alone. Bivariate mapping was performed both using a test for a common marker effect (‘common’) and a test whether there is any marker effect (‘full’), described in the Materials and Methods (see also [14]).

**Fig 1:**
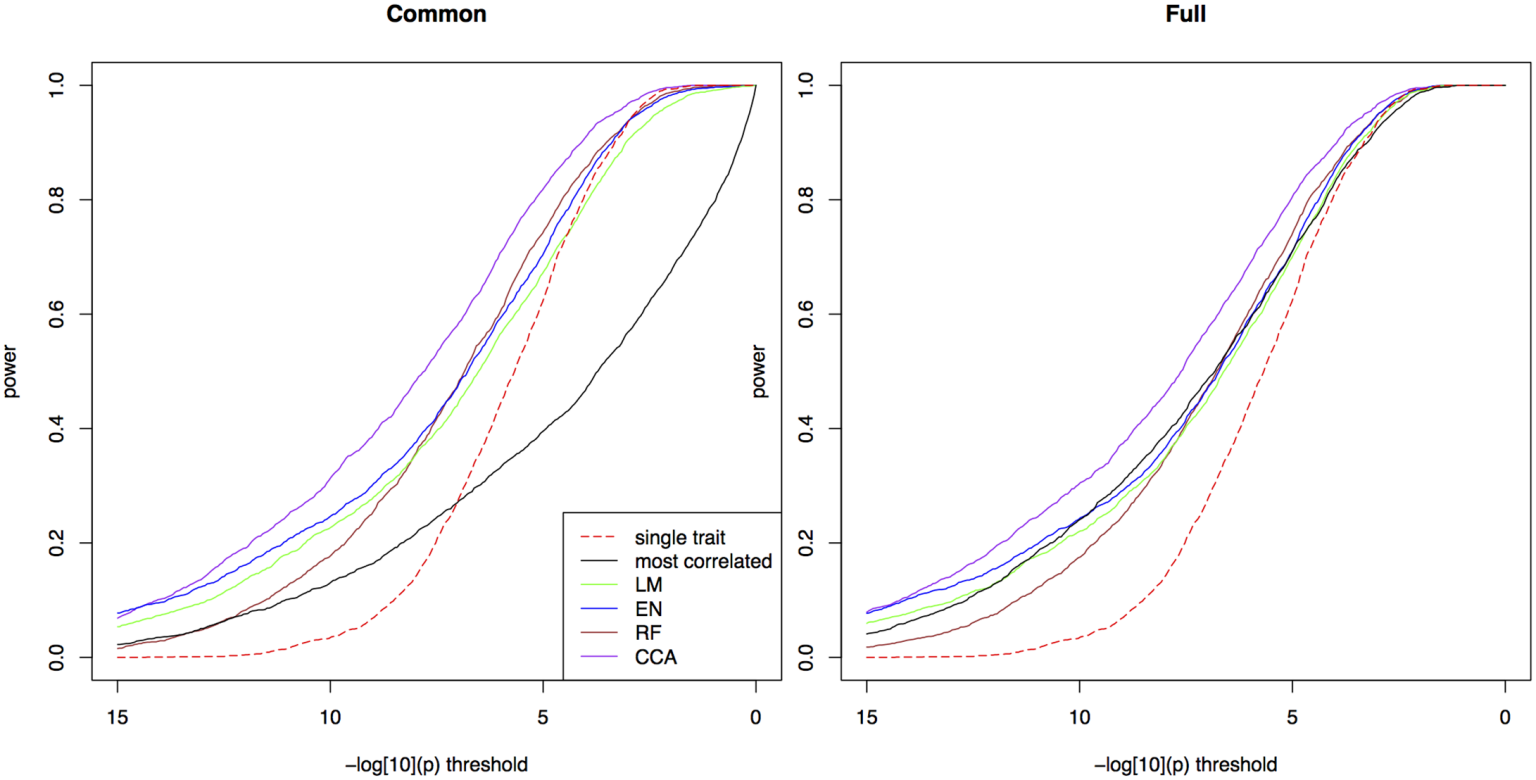
Power in simulations. The proportion of simulated traits for which the -log10(p) value of the causal SNP is above the threshold, for single trait mapping (red), bivariate ETM with the most correlated environmental variable (black), and bivariate ETM with 4 different prediction methods (LM, EN, RF, CCA; respectively green, blue, brown and purple). Bivariate ETM was performed by testing for a common marker effect (left) and by testing whether there is any effect on environment or trait (right). The causal SNP explained 5% of the variance of the simulated trait, while polygenic background and residual variance explained respectively 45% and 50%.

We first simulated a scenario where the heritability is 0.5 and the causative SNP explains 5% of the phenotypic variance; correlations between true and observed environmental variables was set to 0.8. For both types of tests, bivariate ETM using predicted traits shows a clear gain in power over univariate mapping (Fig 1). Bivariate analysis using the environmental variable most correlated to the observed trait performs well in the test for any marker effect, but poorly when testing for a common marker effect, especially at lower significance thresholds. For the four prediction methods the two types of tests perform similarly. Using the test for a common marker effect, CCA showed the highest increase in power (e.g. 0.80 at a −log_10_(*p*) threshold of 5, versus 0.64 for univariate mapping). Other methods perform similarly with power ranging between 0.68–0.73 at the same threshold, and achieving larger gains over univariate mapping at higher −log_10_(*p*) thresholds.

There is a clear relationship across simulated traits between the significance of ETM and correlation between the predicted trait and the simulated true adaptive trait (S1 Fig): ETM is most powerful for simulations where this correlation is large. At lower prediction accuracy the difference with univariate p-values decreases, thus giving smaller differences in power at low −log_10_(*p*) thresholds.

Similar differences between methods are observed in 8 additional scenarios with heritabilities 0.2, 0.5 and 0.8 and the causative SNP explaining 2%, 5% and 10% of the phenotypic variance (S2a–S2i Fig). As expected, the advantage of ETM increases for larger proportions of variance explained. In S2a–S2i Fig we also compared bivariate ETM with univariate mapping on the predicted traits, the latter having lower power for most prediction methods, except for low heritabilities. For CCA, univariate mapping also performs well for higher heritabilities.

Next, we modified the scenario of Fig 1 in the following ways: by lowering the correlations between true and observed environmental variables to 0.5 (S3 Fig), by introducing genetic correlations between the trait and some of the environmental variables (S4 and S5 Figs), and by removing the association between the environmental variables and the causative SNP (S5 and S6 Figs). In the first case, the larger measurement errors in the observed environmental variables leads to a decrease in power of ETM, which however is still more powerful than univariate mapping (S3 Fig). We then performed simulations where the polygenic component of the trait is correlated with the environmental variables defining the true adaptive trait, reflecting the presence of adaptive loci elsewhere on the genome. When the SNP explains 5% of phenotypic variance (as in the main scenario), differences among methods become smaller, in particular between CCA and ETM with the correlated variable (S4 Fig). When the SNP does not affect the phenotype, p-values appear randomly distributed on the unit interval (S5 Fig), indicating that ETM adequately corrects for population structure. In our last scenario (S6 Fig), neither the SNP under consideration nor the polygenic effect was related to the environmental variables. In this case ETM has lower power than univariate mapping, as the SNP is only associated with one the two variables. The largest loss in power then occurs in the test for a common effect, while also the test for any marker effect is affected due to less degrees of freedom [14].

Given the similar performance of the two tests we chose to present all subsequent results for the common marker effect only. We consider this test to be conceptually more appropriate for the detection of loci associated with both the observed trait and its selective environment, which are expected to be positively correlated.

### Environmental prediction of flowering time in Arabidopsis

We used the statistical methods described above to predict flowering time variation among 149 *Arabidopsis thaliana* accessions [1], using public data for 61 environmental variables (S1 File). These predictions will be used in bivariate and univariate ETM below. As expected [23] [17], flowering time is strongly correlated with variables related to latitude such as day length, potential evapotranspiration and temperature (S7 Fig). Spring and summer day length are most correlated with flowering time [7], each explaining 40% of variation compared to 29% for latitude itself. The importance of these variables is reflected in the trait predictions (S8–S11 Figs), where day length is among the most important variables for all prediction methods. The contribution of other variables varies between methods, with the LM and RF prediction assigning relatively high importance to precipitation variables not strongly correlated with latitude (S8 and S10 Figs). The highest correlation between the predicted trait and any single environmental variable, summer day length in all cases, ranges between 0.71–0.84 for LM, RF and CCA but is notably higher for EN (*r* = 0.98) (S8–S11 Figs). The EN-predicted trait may therefore offer little advantage over day length when used in bivariate ETM. Notwithstanding the differences between methods, trait predictions are highly correlated among themselves (*r* = 0.78–0.88) and with the observed trait (*r* = 0.84 (CCA) to *r* = 0.68 (EN)), suggesting that ETM performance will be similar for different prediction methods.

### Bivariate ETM for flowering time

**Fig 2:**
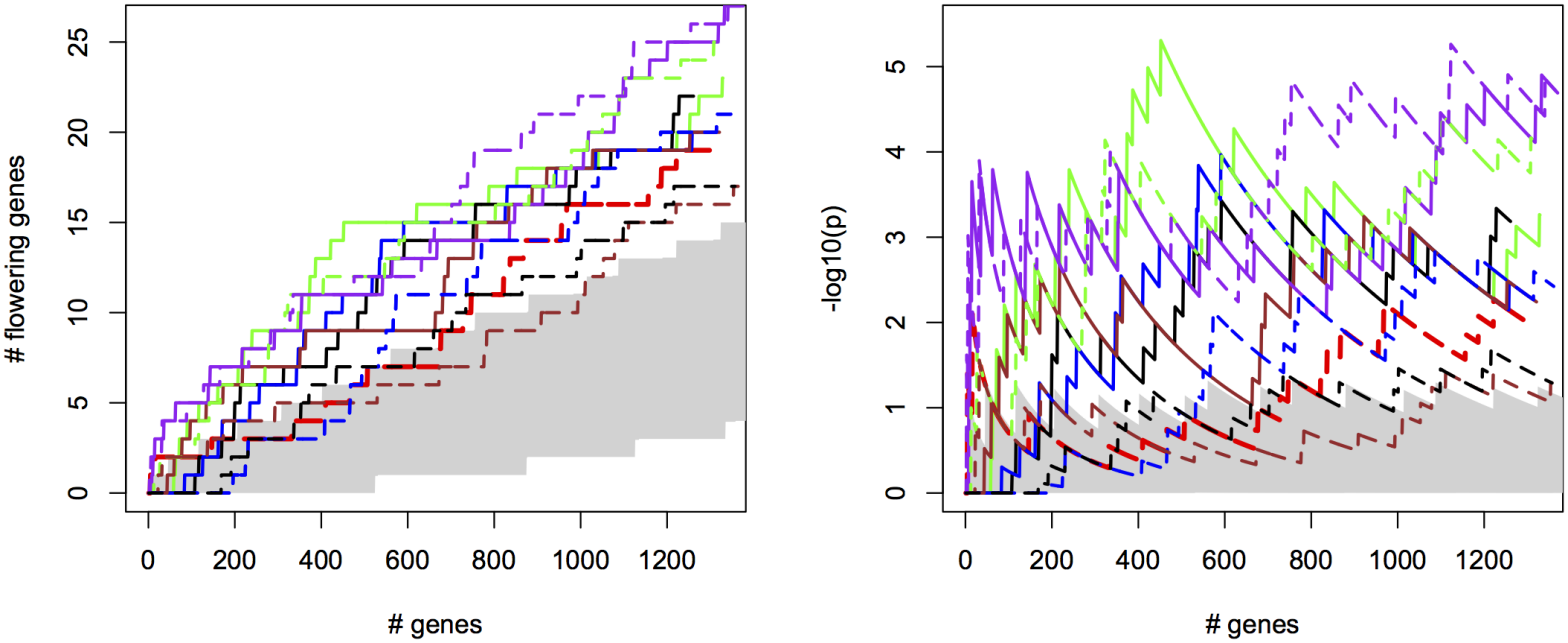
Bivariate ETM for flowering time in *A. thaliana*, for different prediction methods (solid lines) and univariate analysis using observed flowering time, the most correlated environmental variable and the different trait predictions (dotted lines). Left panel: number of known flowering genes recovered, as a function of total number of genes considered. Right panel: corresponding enrichment probabilities (-log10(p)). The following methods were used: CCA (purple), LM (green), RF (brown), EN (blue), analysis using summer day length and observed flowering time are marked in black and red respectively. Enrichment is defined as the probability of recovering *k* out of *m* genes by chance, under the hypergeometric distribution. The area in gray marks the 5% upper and lower percentiles based on 200 permutations of the univariate/bivariate traits.

For the different methods, we measured the cumulative success in recovering 240 known flowering genes (S2 File) as a function of the number of evaluated candidate genes. We thereby assume that GWAS results are used to create a list of candidate SNPs or genes of a certain length as a basis for further evaluation (see S12 Fig for recovery as a function of p-values for comparison). SNPs were sorted by increasing p-value and candidates were defined as genes overlapping with or being closest to any of the top 2000 SNP positions, evaluated successively in order of significance (approximately 1% of all SNPs). We compared univariate association mapping on observed flowering time, bivariate ETM and bivariate analysis using the most correlated trait (Summer day length). Significance of enrichment was calculated as the probability of recovering the observed number of flowering genes by chance (see Materials and Methods). All methods result in significant enrichment but recover only a modest number of genes, yielding 27 flowering genes at most (Fig 2, left). Maximum significance of enrichment ranged from 5 ⋅ 10^−3^ to 4.9 ⋅ 10^−6^ and was achieved after evaluating varying numbers of genes (Fig 2, right). Bivariate ETM outperforms univariate trait mapping over the entire range, with a maximum difference in recovery of 9 flowering genes at 621 evaluated genes (Fig 2, left). ETM based on LM and CCA trait prediction performs particularly well, with high and sustained recovery and peaks of maximum significance of enrichment of 4.9 ⋅ 10^−6^ and 1.3 ⋅ 10^−5^ respectively. Overall, the recovery curves for EN prediction and summer day length are similar, as expected based on the high correlation between the two variables.

For all prediction methods ETM p-values showed some inflation, which also occurred in univariate mapping of the predicted traits, the individual environmental variables and to a lesser extent the observed trait (S13–S14 Figs), and therefore does not appear to be an artifact of our method. Inflation largely disappeared in univariate analyses with a multi-locus mixed model [24](S15 Fig), suggesting that inflation is due to large effects of a small number of loci, insufficiently captured by the kinship matrix.

Considering the top 400 candidate genes for each method, univariate mapping on observed flowering time recovers 2 flowering genes within the first 16, with probabilities of 7.2 ⋅ 10^−3^, but the total of 4 recovered genes does not represent a significant enrichment (*p* = 4.1 ⋅ 10^−1^). Bivariate ETM, by contrast, recovers 9–13 flowering genes within the first 400 candidates (*p* = 5.6 ⋅ 10^−3^ − 2.6 ⋅ 10^−5^), with all prediction methods providing higher enrichment than summer day length (7 genes, *p* = 4.5 ⋅ 10^−2^). The four types of bivariate ETM all recover the genes *SVP*, *GA1*, *DFL2*, *LDL1*, *SPA2*, *FPF1*, *DOG1*, within the first 400 candidates (Table 1). The latter four genes are only recovered by univariate mapping after considering at least 100 additional genes.

**Table 1:** List of genes recovered by different types of bivariate ETM, containing all flowering genes assigned to any of the 2000 SNPs with the lowest p-value for each method. Numbers indicate the rank for each gene (1 being the gene with lowest associated p-value). un: univariate analysis on flowering time, dl: bivariate analysis (MTMM) using summer day length and flowering time.

gene	name	LM	EN	RF	CC	un	dl
AT4G11280	*ACS6*	621	826	760	217	-	1228
AT3G49700	*ACS9*	1223	-	-	-	-	-
AT4G22950	*AGL19*	-	-	-	-	-	1070
AT4G35450	*ARK2A*	347	539	351	913	1187	529
AT4G32980	*ATH1*	240	411	681	-	-	313
AT2G31650	*ATX1*	372	162	44	1007	747	117
AT5G37260	*CIR1*	1253	-	-	1018	-	-
AT2G23380	*CLF*	-	-	-	1319	-	-
AT2G33540	*CPL3*	788	1035	-	1333	728	-
AT4G00450	*CRP*	387	449	-	353	332	562
AT2G38050	*DET2*	374	-	752	564	-	752
AT4G03400	*DFL2*	59	171	97	8	5	108
AT5G45830	*DOG1*	90	347	60	63	507	504
AT4G03430	*EMB2770*	208	343	694	143	837	213
AT4G15880	*ESD4*	-	-	1028	-	-	-
AT5G01400	*ESP4*	-	-	-	-	-	974
AT1G04400	*FHA*	-	518	888	-	-	485
AT2G21070	*FIO1*	-	-	-	-	868	-
AT5G10140	*FLC*	-	-	-	-	671	-
AT5G24860	*FPF1*	117	235	134	15	677	990
AT1G03160	*FZL*	422	535	664	836	-	412
AT4G02780	*GA1*	220	84	78	36	16	170
AT1G62830	*LDL1*	161	360	218	355	956	439
AT3G18165	*MOS4*	-	-	-	1201	-	-
AT2G44170	*NMT2*	-	-	834	-	-	-
AT2G43010	*PIF4*	-	-	-	542	-	-
AT2G18790	*PHYB*	-	943	-	1078	-	-
AT3G12810	*PIE1*	-	-	-	668	-	-
AT1G09530	*PIF3*	-	1258	-	-	-	757
AT3G62090	*PIL2*	-	-	922	-	-	-
AT1G64520	*RPN12a*	-	-	-	1160	-	-
AT3G52180	*SEX4*	1274	830	-	-	-	1212
AT4G11110	*SPA2*	63	257	364	11	968	200
AT2G42200	*SPL9*	-	-	-	848	-	-
AT1G16610	*SR45*	853	-	-	1099	-	-
AT3G28730	*SSRP1*	452	591	685	966	-	592
AT4G02700	*SULTR3;2*	-	-	1253	-	410	1215
AT5G19600	*SULTR3;5*	1328	-	-	-	-	-
AT2G22540	*SVP*	72	126	173	139	147	198
AT3G22380	*TIC*	1041	-	-	292	466	-
AT4G20370	*TSF*	-	-	-	1089	1157	-
AT5G57380	*VIN3*	-	-	-	-	1222	-
AT5G07200	*YAP169*	-	-	-	-	822	-

**Fig 3:**
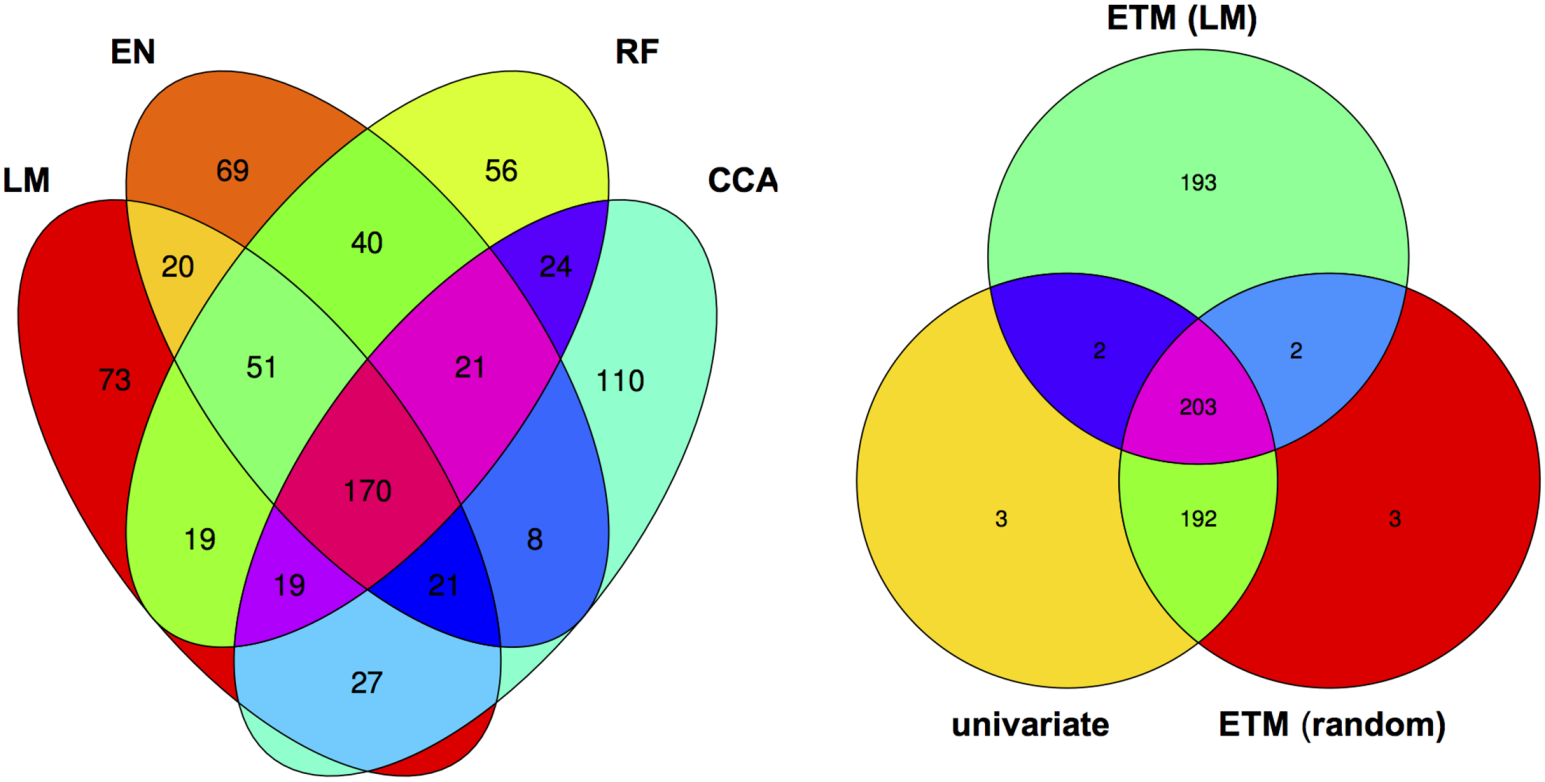
Venn diagram of the top 400 candidate genes. Left panel: overlap between genes identified by bivariate ETM using the four different prediction methods. Right panel: overlap between univariate association analysis using observed flowering time, bivariate ETM with observed and predicted (LM) flowering time, bivariate ETM with observed flowering time and a randomly simulated variable correlated (*r* = 0.8) to observed flowering time.

Although different bivariate ETM analyses identify different sets of genes, overlap is relatively high. Considering the top 400 candidate genes of each prediction method, an average of 249 (220–282) genes is shared between prediction methods (Fig 3), compared to an average of 199 between bivariate ETM and univariate mapping. Bivariate ETM and standard association mapping thus recover different genes. These differences are unlikely to be due to chance, as shown by the fact that bivariate ETM (LM prediction) with a simulated trait equally correlated with the observed trait (i.e. *r* = 0.81) identifies only 5 unique genes compared to univariate association mapping (Fig 3).

### Mapping genes with incomplete phenotypic data using univariate ETM

**Fig 4:**
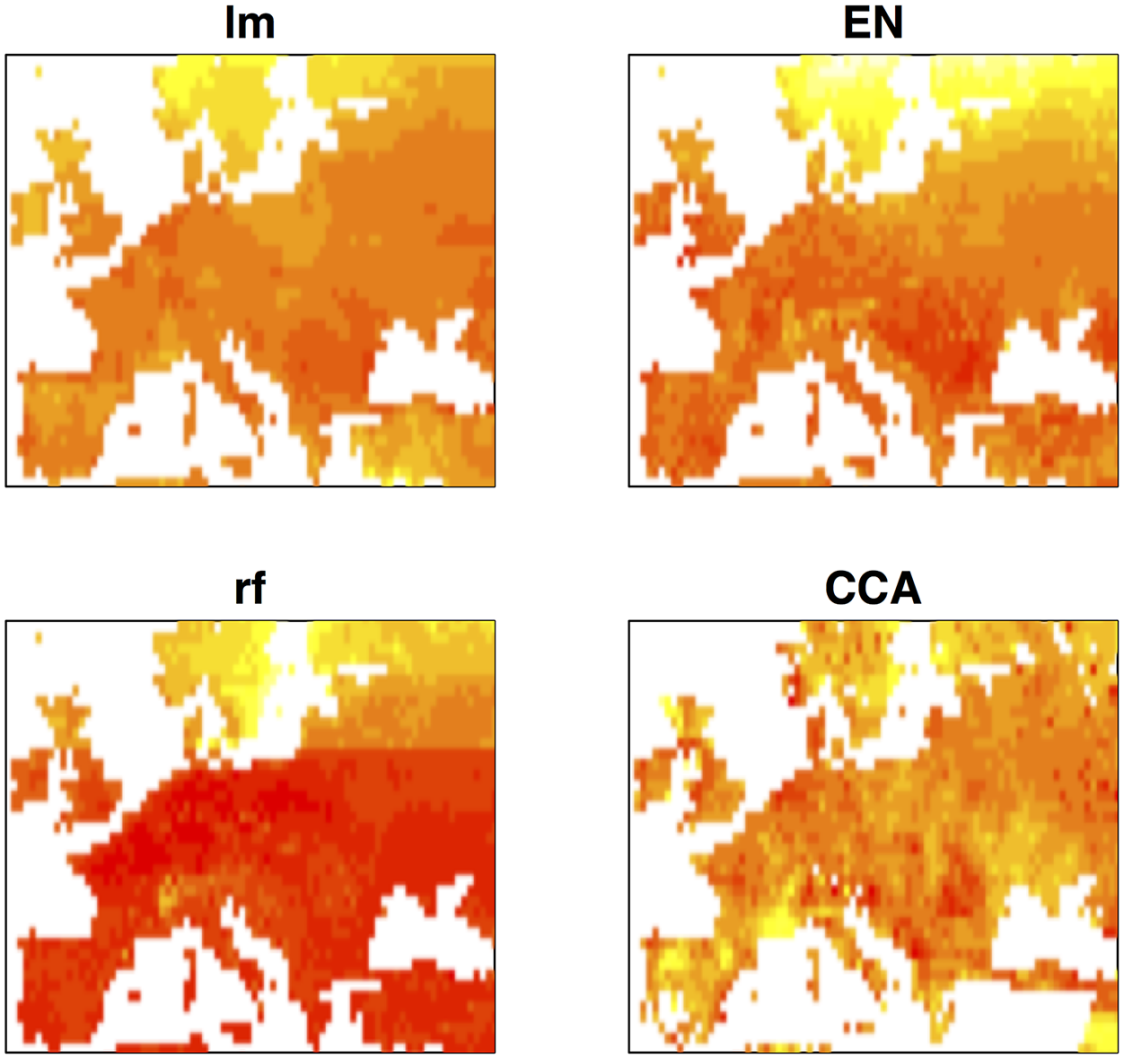
Geographic maps of predicted trait values for the different prediction methods. Heatmaps showing low to high values of predicted (scaled) flowering time as red (early) to yellow (late).

Environmental prediction of trait values offers the possibility of association mapping when phenotypic data is incomplete. Traits of interest can be predicted across geographic space using geographic information and association mapping may then be performed on any set of georeferenced individuals for which genotypic data are available. Fig 4 shows geographic maps of predicted flowering time obtained by the four different prediction methods. Although the importance of latitude is evident, in all cases the predicted trait surface clearly reflects the effect of variables that are not strongly correlated with latitude. We compared the performance of univariate ETM to that of (univariate) association mapping on summer day length and latitude, for a dataset of 478 genotyped and georeferenced accessions for which no flowering time data was available and whose range of predicted trait values did not exceed that observed for the 149 phenotyped individuals.

**Fig 5:**
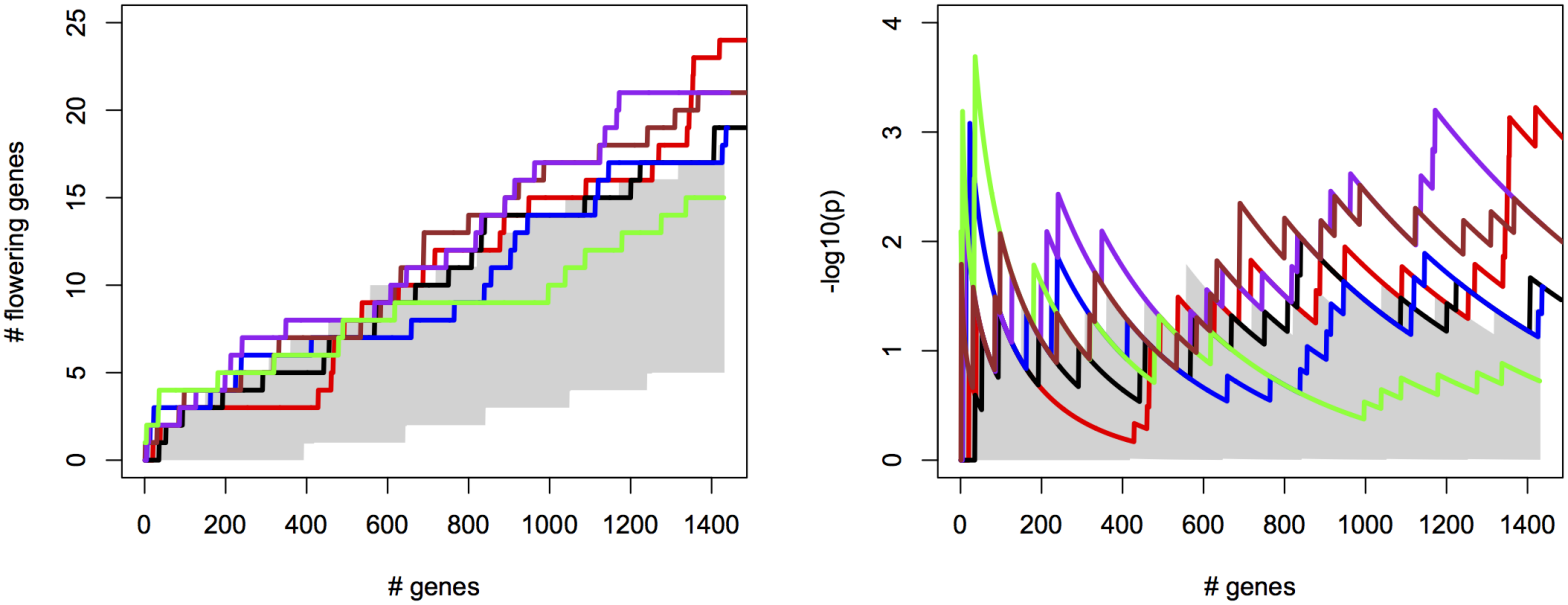
Univariate ETM for flowering time in *A. thaliana*, for different prediction methods, compared to univariate mapping of summer day length and latitude. Left panel: number of known flowering genes recovered, as a function of total number of genes considered. Right panel: corresponding enrichment probabilities (-log10(p)). Enrichment is defined as the probability of recovering *k* out of *m* genes by chance, under the hypergeometric distribution. Colors represent ETM with CCA prediction (purple), ETM with LM (green), ETM with RF (brown), ETM with EN (blue), univariate mapping of latitude (black), univariate mapping of summer day length (red). Recovery and enrichment based on randomly sampled SNPs are shown as reference (grey dashed line). The area in gray marks the 5% upper and lower percentiles based on 200 permutations of the univariate/bivariate traits.

**Fig 6:**
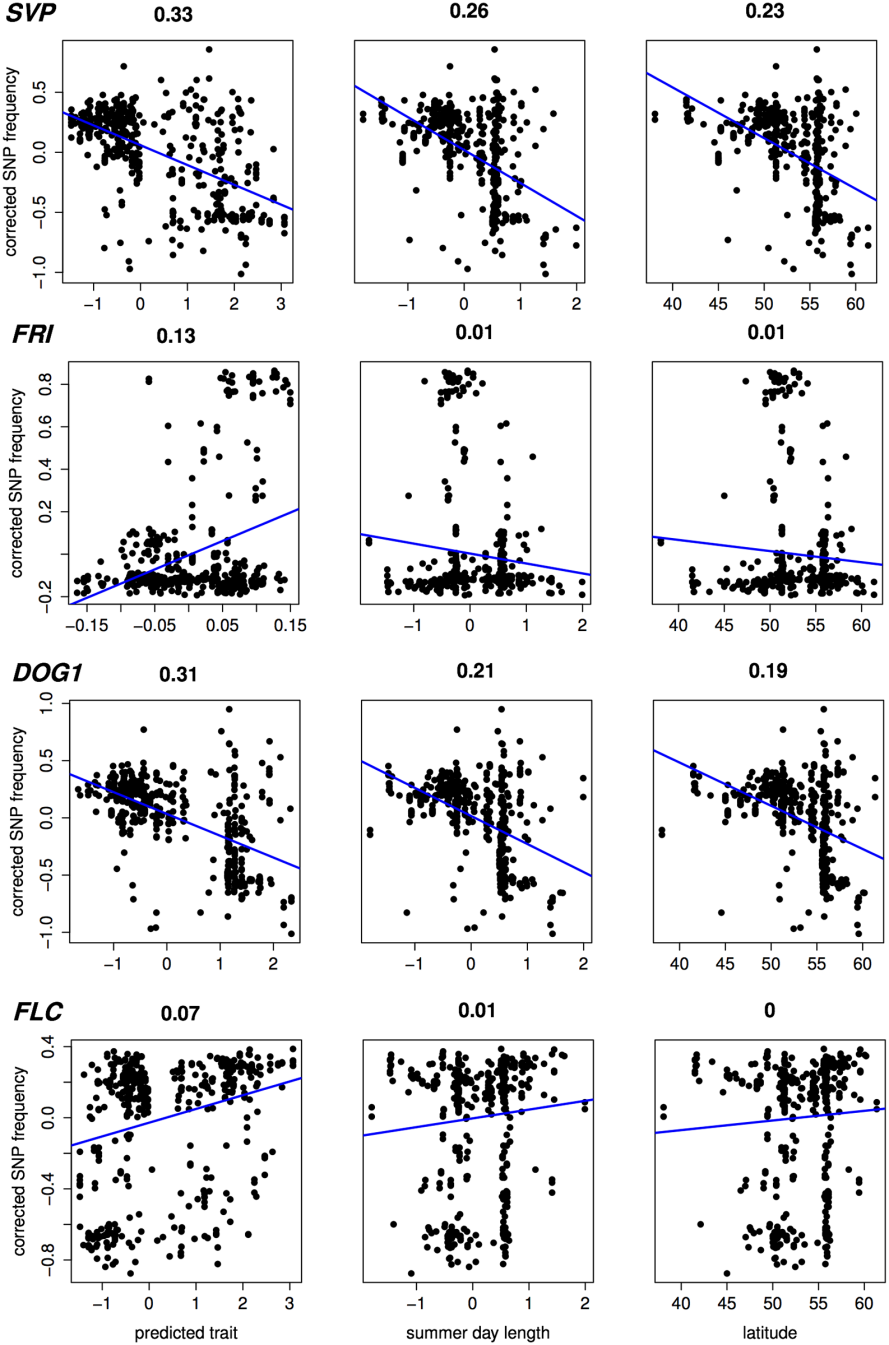
Correlation (Pearson *r* ^2^) with estimated SNP frequency at 4 important flowering loci. Scatter plots showing (structure corrected) SNP frequencies against predicted flowering time, summer day length and latitude, for 478 accessions without phenotypic observations. In case of predicted flowering time, the prediction method yielding the highest significance is shown for each gene (from top to bottom: RF, CCA, LM, RF). Estimates of SNP frequency were obtained using the program *SCAT* [43].

Recovery of known flowering genes is somewhat lower compared to bivariate ETM (Fig 5). Although performance is only slightly higher compared to random permutations, maximum enrichment is significant in all cases. Differences in performance between methods are small, but ETM has higher recovery and enrichment within the first 400 genes compared to mapping the two environmental variables individually. Within these top 400 candidates, *SVP*, *CRP*, *SPA2*, *DOG1*, *PIE1* and *FRI* are recovered by more than one method (Table 2) and for each, ETM with LM prediction requires fewer candidate genes to be evaluated compared to mapping the two environmental variables, although the best prediction method differed between genes. *FRI* is a well studied, major flowering locus in *A. thaliana*[23] [25], which together with the gene *FLC* affects the latitudinal cline in flowering time [26] [17] [27] [28]. *FLC* ranks 617 and 627 using RF and day length respectively, but is not recovered at all by latitude. The relatively weak recovery of *FLC*, *FRI*, *SVP* and *DOG1* with latitude is surprising since all have been reported to show allelic variation with latitude [29]. This suggests that predicted traits used in ETM may be better correlated with the underlying gene frequency at these loci than latitude itself. We confirmed this by estimating the geographic frequencies of the SNP distinguishing the two functional haplotypes at *FLC* and *FRI* [16] and of the SNPs with the lowest p-values at *SVP* and *DOG1*, and correlating these to the different variables including latitude (Fig 6). In each case, the best trait prediction (i.e. yielding highest *r* ^2^ with SNP frequency) has a higher correlation with SNP frequency than either summer day length or latitude. In fact, our data provides no evidence for a latitudinal trend for either *FRI* or *FLC*, while the correlation with predicted flowering time is weak but significant (*p* < 1 ⋅ 10^−9^).

**Table 2:** List of genes recovered by different types of univariate ETM, containing all flowering genes assigned to any of the 2000 SNPs with the lowest p-value for each method. Numbers indicate the rank for each gene (1 being the gene with lowest associated p-value). dl: univariate analysis using summer day length. lt: univariate analysis using latitude.

gene	name	LM	EN	RF	CC	dl	lt
AT4G11280	*ACS6*	618	-	-	-	469	575
AT2G45660	*AGL20*	-	-	-	-	1340	1224
AT4G36920	*AP2*	-	-	-	832	-	-
AT5G24470	*APRR5*	1088	659	-	-	-	-
AT3G10185	AT3G10185	-	-	-	-	466	443
AT1G18450	*ATARP4*	1276	-	-	213	-	-
AT3G51780	*AtBAG4*	-	-	-	1172	-	-
AT1G50960	*ATGA2OX7*	-	-	-	-	1420	-
AT3G63010	*ATGID1B*	-	-	-	647	-	-
AT4G32980	*ATH1*	-	-	986	-	-	-
AT5G03790	*ATHB51*	-	-	-	-	1356	1406
AT3G03090	*AtVGT1*	-	-	690	568	1350	-
AT2G31650	*ATX1*	997	1146	890	-	-	-
AT2G33540	*CPL3*	-	946	-	-	949	-
AT4G20910	*CRM2*	-	1436	-	-	688	808
AT4G00450	*CRP*	5	1120	85	11	-	-
AT5G03730	*CTR1*	-	-	-	890	-	-
AT1G12610	*DDF1*	-	-	-	-	-	832
AT1G63030	*DDF2*	-	-	319	-	-	-
AT4G03400	*DFL2*	-	-	-	349	-	-
AT5G45830	*DOG1*	1	23	31	87	21	36
AT5G62640	*ELF5*	-	-	689	-	888	1201
AT4G03430	*EMB2770*	-	-	-	1166	-	-
AT5G11530	*EMF1*	479	-	-	-	-	-
AT1G04400	*FHA*	-	-	-	-	-	456
AT5G10140	*FLC*	-	839	617	963	627	-
AT3G10390	*FLD*	-	-	-	-	429	292
AT4G00650	*FRI*	181	14	332	5	493	1087
AT5G63980	*FRY1*	-	-	-	-	878	840
AT1G03160	*FZL*	-	-	-	914	-	-
AT4G02780	*GA1*	-	-	1367	-	-	-
AT1G80340	*GA4H*	-	-	-	-	1090	669
AT1G22770	*GI*	-	-	583	-	-	-
AT2G39810	*HOS1*	-	-	-	818	-	-
AT1G09700	*HYL1*	-	856	-	-	-	-
AT5G67100	*ICU2*	-	163	-	-	1270	-
AT3G18165	*MOS4*	-	-	-	-	461	193
AT4G24020	*NLP7*	-	915	-	-	-	-
AT5G48150	*PAT1*	490	-	-	-	1354	1407
AT3G28860	*PGP19*	1337	-	-	-	-	-
AT2G18790	*PHYB*	1039	765	924	-	-	-
AT4G16250	*PHYD*	-	-	633	-	537	568
AT3G12810	*PIE1*	320	225	98	-	-	-
AT3G62090	*PIL2*	-	904	238	-	-	-
AT2G01570	*RGA1*	-	-	-	127	-	-
AT2G47310	*simtoFCA*	-	-	-	199	-	-
AT5G46910	*simtoREF6*	-	1113	-	-	-	-
AT4G33280	*simtoVRN1*	-	-	1310	-	-	-
AT4G11110	*SPA2*	34	239	534	745	91	94
AT3G28730	*SSRP1*	-	-	-	608	-	-
AT4G02700	*SULTR3;2*	-	-	-	1137	1254	-
AT1G23090	*SULTR3;3*	-	412	-	-	1345	-
AT2G22540	*SVP*	36	6	2	241	39	53
AT3G22380	*TIC*	-	1427	1123	-	-	-
AT1G17110	*UBP15*	-	-	800	-	-	-
AT4G16845	*VRN2*	1179	-	-	-	717	751
AT5G57360	*ZTL*	-	-	1242	1125	-	-

## Discussion

We have explored the use of environmentally predicted traits for genome-wide mapping of genes underlying adaptive trait variation. This is basically an extension of the concept of phenotype to include the environment. That idea is not new, in the sense that it has been implicit in most studies relating environment to gene frequency. The novelty of our approach lies in the fact that this extension is made explicit and is used in conjunction with the observed trait of interest to obtain a better approximation of the selection gradient responsible for trait variation. Although this may seem counter-intuitive at first, its merit becomes apparent when considering that information on correlated environmental variables can be used to reduce the effect of experimental error in the same way as correlated traits can [30] [14] [31]. We thereby take advantage of so-called latent variables, which are factors indirectly related to the trait of interest and that are generally considered a source of spurious associations [32].

Although selective forces determining trait variation may sometimes shape allele frequencies at non causal loci (e.g. those affecting an unmeasured adaptive trait), independent estimates of these selective forces can at the same time help to find true associations, particularly when combined with the trait itself. Bivariate ETM is designed to detect genes whose frequencies correlate with selective forces that have shaped a trait of interest. These are likely to affect the target trait directly, although they may also be genes affecting correlated adaptive traits. In our case an average of 87% of the top 2000 SNPs for bivariate ETM had p-values below 0.05 for flowering time itself. Since our primary aim is to find genes related to adaptation however, any gene that is affected by the same selective environment is of interest, regardless of its causal relation to the trait.

The success of this approach does require that traits and the environment provide complementary estimates of underlying selective forces, something that may not always be the case. The result that enrichment for known flowering genes is higher for bivariate ETM than for univariate mapping on the trait itself, and that this is not observed for randomly simulated variables with the same correlation to the observed trait, suggests that predicted and observed traits indeed complement each other. One thing to observe, is that our definition of recovery as the closest gene to a detected SNP, deviates from Atwell et al.’s decision to consider SNPs within 20kb of their candidate genes [1]. Our criterion was chosen to avoid calling multiple genes per evaluation position and reflects the fact that in the Arabidopsis genome, LD is estimated to decay within 10kb on average [33].

Another application of environmental trait prediction is the mapping of adaptive genes in individuals with missing phenotypic information. It offers potential for mining the growing genomic data available for many species without the need for complete phenotypic data, and exploiting the wealth of publicly available geographic and environmental data. Our results on mapping flowering genes in unphenotyped individuals are encouraging in the sense that more genes are found than expected at random. On the other hand, the improvement achieved over single environmental variables such as latitude is rather modest. This probably reflects the fact that environmental variables related to latitude are the dominant selective agents affecting flowering time, making it hard to improve over the use of well chosen single environmental variables. Nonetheless, at several genes with known association with latitude, estimated gene frequencies are more strongly correlated with predicted flowering time than with latitude. This observation provides evidence that mapping on predicted traits has the potential of producing more relevant association results than single environmental variables chosen a priori.

In conclusion, we have provided evidence that integrating environmental and phenotypic data can improve our ability to map genes of adaptive significance. We have thereby explored several statistical methods for modeling traits as a function of the environment. We do not consider our results conclusive with respect to the best prediction method and more work remains to be done in that respect. Alternatives such as sparse multivariate methods [34] may be worth exploring. In addition, it is conceivable to integrate prediction into the MTMM step of our approach, and target the combination of environmental variables with the highest *genetic* rather than phenotypic correlation. This however implies an optimization problem for which no algorithms currently seem to be available. Alternatively, bivariate MTMM could be replaced by multivariate MTMM, including all environmental variables individually (as well as the observed trait), but state-of-the art approaches [15] currently cannot perform GWAS on more than 10 traits.

Another issue is that of inflation, which may affect the distribution of p-values in any GWAS study due to confounding of the polygenic background with population structure [35] [36] or the occurrence of large effect loci [24]. Although we adopt the standard MTMM approach of correcting for population structure by a marker-based kinship matrix it is clear that for traits like flowering time there is a certain degree of residual inflation. The fact that inflation for most traits was adequately controlled in a univariate multi-locus mixed model (MLMM), suggests there is scope for the development of a multi-locus version of MTMM.

In terms of application, it will be interesting to test the added value of our approach for traits that are more weakly correlated with known environmental factors, such as is the case for disease or drought resistance. We hope that the present work may serve as a first step in moving adaptation mapping beyond the traditional univariate analysis of traits and environmental variables and towards a more integrated use of all available data.

## Materials and Methods

### Arabidopsis data

We used two datasets from two highly cited examples of trait association and environmental association in *A. thaliana* [1] [7]. The first set consisted of 199 phenotyped accessions of which we retained 149 individuals with available Eurasian geographic coordinates and no missing data for any of the included traits. We reduced data on flowering time measured at 10, 16 and 22 degrees Celsius to a single principal component explaining 90 percent of total variation, which was used in all subsequent analyses, unless stated otherwise. The second set consisted of 948 georeferenced accessions, sampled across Eurasia, of which we excluded 39 accessions with non-terrestrial coordinates.

High-density Single Nucleotide Polymorphism (SNP) data, using the Affymetrix 250K SNP-tiling array was available for both studies [29]. SNP positions and gene annotations were based on version 10 of the Arabidopsis genome annotation (TAIR10). A list of 240 mapped candidate genes for flowering time was obtained from [1] and [37], complemented with a subset of genes derived from the list of known Arabidopsis flowering genes available from the Prof. Coupland lab (MPIPZ, Cologne, Germany; https://www.mpipz.mpg.de/14637/Arabidopsis_flowering_genes). SNP positions with the highest frequency differentiation at functional variants of the flowering genes *FLC* and *FRI* were identified based on 85 accessions for which functional haplogroups were available [16].

### Environmental data and analyses

We compiled georeferenced climatic, soil and vegetation data from a variety of public sources (S1 File), resulting in a final set of 61 environmental variables with a spatial resolution ranging from 0.5 to 50 km. Remote sensing data were mosaicked, time averaged and converted to GIS raster layers with custom R scripts, using functions from the programs *cdo* [38], *MRT* [39] and the package *Raster* [40]. Average day length for different seasons was calculated from latitude [41]. Visualization of geographic data and assignment of environmental variables to sample locations was done using the *QGIS* software [42]. Estimates of continuous allele frequencies across the landscape were produced using the program *SCAT* [43].

### Environmental Trait Mapping (ETM)

Our ETM procedure can be summarized as follows. First we predict the observed phenotype as a function of environmental data. Below we describe four possible prediction methods, but in principle any method can be used here. Provided this prediction is not constant we then perform bivariate GWAS on the observed and predicted phenotype (bivariate ETM), or univariate GWAS on the predicted phenotype alone (univariate ETM). In the case of bivariate ETM, we consider the test for a common marker effect (details given below), but the test for any marker effect is possible as well.

#### Modeling traits using environmental data

We made environmental trait predictions using standard linear regression with backward selection (LM), elastic nets (EN), random forests (RF) and canonical correlation analysis (CCA), which we describe below. Suppose that for accessions *i* = 1, …, *n* we have observations (*y*_*i*_, *x*_1,*i*_, …, *x*_*p*,*i*_) on the standardized phenotype *y* and standardized environmental predictors *x*_1_, …, *x*_*p*_. Let *X* = [*x*_1_ … *x*_*p*_] be the *n* × *p* predictor matrix, and let $X_{i}\in R^{p}$ (*i* = 1, …, *n*) denote its rows.

For most of our examples, between 10 and 50 predictors are available. This number is smaller than the number of accessions, but to avoid over-fitting and allow for interactions, some form of variable selection is desirable. LM, elastic nets and random forests select, in different ways, the environmental variables most relevant for prediction. CCA does not perform variable selection. In the case of elastic nets and random forests we also include the second moments $x_{1}^{2},\ldots ,x_{p}^{2}$ and the first order interactions *x*_*j*_ *x*_*j*′_, 1 ≤ *j* < *j*′ ≤ *p*, giving in total 2*p* + *p*(*p* − 1)/2 predictors. Since this number exceeds the sample size, we did not include interactions for LM and CCA. For ease of notation, we will now assume that *p* is the total number of predictors (either the total number of variables (in case of LM and CCA) or the total number of variables plus the number of interactions (EN and RF)).

Linear regression with backward selection assumes the linear model *y* = *X*_(*S*)_ *β*_(*S*)_, where *X*_(*S*)_ is *X* restricted to a certain subset *S* ⊂ {1, …, *k*} of environmental variables, and *β*_(*S*)_ the corresponding vector of regression coefficients. We start with the complete set of predictors contained in *X* and then perform backward selection using Akaike’s information criterion (AIC [44]), using the R-function stepAIC. The final model is that with the lowest AIC. Note that since both *y* and the columns of *X* are standardized, no intercept is contained in the model.

In case of elastic nets [20], variable selection is achieved by penalized likelihood, and the regression coefficients are estimated by

$$\hat{\beta }=\underset{\beta }{\mathrm{argmin}}\{\frac{1}{\sigma^{2}}\sum_{i=1}^{n}{\left(y_{i}-X_{i}\beta \right)}^{2}+\lambda \;(\frac{1-\alpha }{2}\sum_{j=1}^{p}\beta_{j}^{2}+\alpha \sum_{j=1}^{p}\left|\beta_{j}\right|)\},$$

where *λ* > 0 is the amount of penalization and *α* ∈ [0, 1] determines the weight of the *L*_1_- and *L*_2_-penalty. The term $\sum_{j=1}^{p}\left|\beta_{j}\right|$ forces some coefficients *β*_*j*_ to be exactly zero. We fixed *α* = 0.5 and chose *λ* by 10-fold cross-validation. We used the implementation in the R-package glmnet [45].

Random forests, introduced in [21], combine bagging (bootstrap aggregation; [46]) and tree based methods. Although initially used for classification, random forests are now also commonly used for prediction. A large number (e.g. 500) of bootstrap samples is drawn, and for each bootstrap sample a regression tree is ‘grown’. This tree recursively divides the predictor space in hypercubes.

Finally, we used canonical correlation analysis (CCA), which is particularly appealing when several correlated traits *y* are available. CCA constructs pairs of linear combinations of *y*’s and *x*’s (canonical variables) that have maximal correlation with one another. We then model the traits by the linear combination of environmental predictors from the first pair of canonical variables. In case of the Atwell *et al*. data the three flowering traits measured at 10, 16 and 22 degrees Celsius were included, and the linear combination from the first pair of canonical variables was subsequently used in bivariate ETM. In the simulations CCA was performed with a single trait.

LM, EN, RF and CCA all model the observed phenotype as a function of environmental predictors, using accessions for which both phenotypic and environmental data are available. If there is a second set of accessions with only environmental data, predictions are made using the model obtained using the accessions with complete data. In case of LM and EN for instance, the vector of estimated regression coefficients $\hat{\beta }$ gives predictions $X_{i^{\prime }}\hat{\beta }$, *X*_*i*′_ containing the environmental predictors for accession *i*′ with missing phenotypic data.

#### Genome wide association analysis

We adopt the multi-trait mixed model (MTMM) framework of [14] and [15], which allows for both genetic and environmental correlations between traits. Marker effects are modeled as a combination of effects that are common to both traits and trait-marker interaction effects. Following the notation of [14], it is assumed that

$$[\begin{array}{c} y_{1}\\ y_{2} \end{array}]=s_{1}\mu_{1}+s_{2}\mu_{2}+x\beta +\left(x\times s_{1}\right)\alpha +v,$$ (1)

where *s*_*i*_ is the column vector which is one for observations on trait *i* and zero otherwise, and *x* is the vector of 2*n* marker scores. As in [14], we have trait-specific means *μ*_1_ and *μ*_2_, and a Gaussian vector *v* with covariance

$$Cov\left(v\right)=(\begin{array}{cc} \sigma_{g,1}^{2}K & \rho_{g}\sigma_{g,1}\sigma_{g,2}K\\ \rho_{g}\sigma_{g,1}\sigma_{g,2}K & \sigma_{g,2}^{2}K \end{array})+(\begin{array}{cc} \sigma_{e,1}^{2}I_{n} & \rho_{e}\sigma_{e,1}\sigma_{e,2}I_{n}\\ \rho_{e}\sigma_{e,1}\sigma_{e,2}I_{n} & \sigma_{e,2}^{2}I_{n} \end{array}),$$ (2)

given a *n* × *n* genetic relatedness matrix *K*. The environmental correlation *ρ*_*e*_ is only to be included in the model if both traits are measured on the same individuals. In the present context, we consider an observed and a predicted trait. Since the latter is a function of the observed trait and environmental data, we considered including *ρ*_*e*_ in the model. This however resulted in either numerical instabilities or estimates *ρ*_*e*_ close to zero; we therefore dropped *ρ*_*e*_ from the model. The marker under investigation has a common effect *β* on both *y*_1_ and *y*_2_. The effect *α* is specific to the first trait. Following [14], the following tests were performed for each marker:

1. for any genetic effect (‘full’): the full model against the null-model (*α* = *β* = 0).
2. for a common genetic effect (‘common’): the model with *α* = 0 against the null-model (*α* = *β* = 0).

[14] also proposed a test for trait-specific effects (the full model against the model with *α* = 0), which will not be considered here. In Eqs (1) and (2) we assumed that for each individual, observations on the two traits are available. It is however straightforward to extend the model to situations with disjoint or only partially overlapping sets of individuals (see [14], Supplementary note).

### Analysis of flowering gene recovery

For all methods (bivariate/univariate ETM, univariate mapping) SNPs were ordered by their significance and the 2000 SNPs with lowest p-values were considered as candidate SNPs. We assigned each of these SNPs to the gene(s) overlapping with its position or to the closest gene in the case of non-genic SNPs. This criterion differs from that used by Atwell et al. (2010) [1], who assigned genes within a 20kb window around each SNP as candidates. Our criterion was designed to minimize the number of genes evaluated per SNP, without requiring arbitrary decisions on relevant window size (See S16 Fig for a comparison of results using different criteria).

We counted how many out of the 240 known flowering genes were recovered as a function of the number of unique genes considered when going down the ordered list of candidate genes. At each point, enrichment was calculated as the hypergeometric probability of finding (at least) the number of unique flowering genes, given the number of genes evaluated so far, the total of flowering genes (240) and the total of 29,477 genes assigned to any of the SNPs.

### Simulations

We simulate traits and environmental variables for a fixed set of *n* = 300 accessions taken from the regmap, of which we randomly selected 100 Swedish, 100 French, 50 German and 50 Czech accessions. Each simulation consists of *k* = 30 simulated environmental variables and 1 simulated trait.

Each simulation starts by drawing a Gaussian *n* × *k* matrix *X*_*T*_, containing the true (unobserved) environmental variables at the locations of origin of the accessions. *X*_*T*_ specifies what we will call the true environment. First we randomly draw a subset *S* ⊂ {1, …, *k*}, containing *s* = 10 environmental variables, which will later form the environmental gradient. We will use the notation *X*_*T*_(*S*) for the submatrix of *X*_*T*_ with columns defined by *S*.

To model confounding with population structure, the variables in *X*_*T*_ contain polygenic components, such that their heritabilities are 0.5. Specifically, *X*_*T*_ is the sum of *G*_*env*_ and *E*_*env*_, which are drawn from zero mean matrix variate normal distributions (see e.g. [15]). *G*_*env*_ is simulated together with the column (*n* × 1) vector *G*_*trait*_, such that (*G*_*env*_, *G*_*trait*_) is matrix variate normal with column covariance matrix *V*_*G*_ and row covariance given by a marker-based kinship matrix *K*. *G*_*trait*_ is the polygenic signal in the observed trait *y*_*O*_ (defined below). *V*_*G*_ is the (*k* + 1) × (*k* + 1) covariance matrix of (*G*_*env*_, *G*_*trait*_). The off-diagonal elements of *V*_*G*_ are chosen such that for each pair of variables in *S*, the genetic correlation is 0.5. Also the genetic correlations between environmental variables from the complement of *S* are set to 0.5, while it is zero for all variables *j* ∈ *S* and *j*′ ∈ *S* ^*c*^. The correlation between *G*_*trait*_ and the columns of *G*_*env*_(*S*) is either 0 or 0.5. In the latter case, this reflects the assumption that *G*_*trait*_ is to a certain extent adaptive. The correlation between *G*_*trait*_ and the columns of *G*_*env*_(*S* ^*c*^) is always 0. The row and column covariance matrices of *E*_*env*_ are both diagonal.

Given the outcome of *X*_*T*_ we then simulate *X*_*O*_, the *observed* environmental variables, by adding random Gaussian errors with variance chosen as to achieve a correlation of 0.80, for each corresponding pair of columns in *X*_*T*_ and *X*_*O*_.

We then define the environmental gradient as *y*_*T*_ = *βX*_*T*_(*S*), where *β*_1_, …, *β*_*s*_ are drawn independently from a uniform distribution on the interval [−1, 1]. For simplicity we assume that *y*_*T*_ is the (unobserved) adaptive phenotype, although more complex relations between environmental gradients and phenotypes can be expected in nature.

The vector *f* of causal allele frequencies at each simulated location, is defined as $f\left(y_{T}\right)=e^{\lambda y_{T}}/\left(1+e^{\lambda y_{T}}\right)$ with *λ* = 3, and hence has a correlation of 1 with *y*_*T*_. A corresponding genotypic vector *g* is formed by sampling a single allele for each location from a Bernoulli distribution with probability *f*. Finally, we simulate the vector of *observed* phenotypes *y*_*O*_ = *β*_*snp*_ *g* + *G*_*trait*_ + *E*_*trait*_, where *β*_*snp*_ represents the SNP-effect on the trait, *G*_*trait*_ is the polygenic effect defined above, and *E*_*trait*_ is residual noise.

We performed the following sets of 2000 simulations:

- The main set (Fig 1), where *β*_*snp*_ and the variance of *E*_*trait*_ are chosen such that the SNP explains 5% of the phenotypic variance, while *G*_*trait*_ and *E*_*trait*_ explain respectively 45% and 50%, i.e. the heritability of the observed trait is 0.5. The correlations between *G*_*trait*_ and *G*_*env*_(*S*) are set to 0.
- In S2a–S2i Fig, we repeated the simulations from the main set, for heritabilities of 0.2, 0.5 and 0.8, and the causal SNP explaining 2%, 5% and 10% of the phenotypic variance.
- In S3 Fig, we repeated the simulations from the main set, lowering the correlations between true and observed variables to 0.5.
- In S4 Fig, we repeated the simulations from the main set, the correlations between *G*_*trait*_ and *G*_*env*_(*S*) being 0.5.
- In S5a and S5b Fig, we repeated the simulations from the main set, the correlations between *G*_*trait*_ and *G*_*env*_(*S*) being 0.5. Additionally, the SNP effect (*β*_*snp*_) was set to 0, and *G*_*trait*_ explained 50% of the variance.
- In S6 Fig, we repeated the simulations from the main set, but sampled the vector *g* of SNP scores randomly from independent Bernoulli(0.5) distributions, i.e. independent of any environmental variable.

In all cases, ETM p-values from simulations yielding constant trait predictions were set to their corresponding univariate GWAS p-values.

## Supporting Information

S1 Fig Simulation results showing the relation between performance gain of bivariate ETM (LM) and the Pearson correlation of the predicted trait and true environmental gradient. The regression line is shown in red and the line marking equal performance of the two methods is marked in blue. Only results for non-constant predictions are shown.
(PDF)

S2 Fig Power in simulations for single trait mapping (red), bivariate ETM with the most correlated environmental variable (black), bivariate ETM with 4 different prediction methods (LM, EN, RF, CCA; respectively green, blue, brown and purple solid lines), and single trait mapping with the 4 predicted traits (same colors, dashed lines). Bivariate ETM was performed by testing for a common marker effect (top) and by testing whether there is any effect on environment or trait (bottom).
(a) *h* ^2^ = 0.2. The causal SNP explained 2% of the variance of the simulated trait, while polygenic background and residual variance explained respectively 18% and 80%. Correlations between true and observed environmental variables were 0.8.
(b) *h* ^2^ = 0.2. The causal SNP explained 5% of the variance of the simulated trait, while polygenic background and residual variance explained respectively 15% and 80%. Correlations between true and observed environmental variables were 0.8.
(c) *h* ^2^ = 0.2. The causal SNP explained 10% of the variance of the simulated trait, while polygenic background and residual variance explained respectively 10% and 80%. Correlations between true and observed environmental variables were 0.8.
(d) *h* ^2^ = 0.5. The causal SNP explained 2% of the variance of the simulated trait, while polygenic background and residual variance explained respectively 48% and 50%. Correlations between true and observed environmental variables were 0.8.
(e) *h* ^2^ = 0.5. The causal SNP explained 5% of the variance of the simulated trait, while polygenic background and residual variance explained respectively 45% and 50%. Correlations between true and observed environmental variables were 0.8.
(f) *h* ^2^ = 0.5. The causal SNP explained 10% of the variance of the simulated trait, while polygenic background and residual variance explained respectively 40% and 50%. Correlations between true and observed environmental variables were 0.8.
(g) *h* ^2^ = 0.8. The causal SNP explained 2% of the variance of the simulated trait, while polygenic background and residual variance explained respectively 78% and 20%. Correlations between true and observed environmental variables were 0.8.
(h) *h* ^2^ = 0.8. The causal SNP explained 5% of the variance of the simulated trait, while polygenic background and residual variance explained respectively 75% and 20%. Correlations between true and observed environmental variables were 0.8.
(i) *h* ^2^ = 0.8. The causal SNP explained 10% of the variance of the simulated trait, while polygenic background and residual variance explained respectively 70% and 20%. Correlations between true and observed environmental variables were 0.8.
(PDF)

S3 Fig Power in simulations (*h* ^2^ = 0.5), correlations between true and observed environmental variables being 0.5. Colors represent single trait mapping (red), bivariate ETM with the most correlated environmental variable (black), and bivariate ETM with 4 different prediction methods (LM, EN, RF, CCA; respectively green, blue, brown and purple). Bivariate ETM was performed by testing for a common marker effect (top) and by testing whether there is any effect on environment or trait (bottom). The causal SNP explained 45% of the variance of the simulated trait, while polygenic background and residual variance explained respectively 45% and 50%.
(PDF)

S4 Fig Power in simulations, the genetic correlation between the observed trait and each of the 10 environmental variables defining the environmental gradient being 0.5. Colors represent single trait mapping (red), bivariate ETM with the most correlated environmental variable (black), and bivariate ETM with 4 different prediction methods (LM, EN, RF, CCA; respectively green, blue, brown and purple). Bivariate ETM was performed by testing for a common marker effect (top) and by testing whether there is any effect on environment or trait (bottom). The causal SNP explained 5% of the variance of the simulated trait, while polygenic background and residual variance explained respectively 45% and 50%.
(PDF)

S5 Fig QQ-plots of −log_10_(*p*) values in simulations without a SNP effect, for single trait mapping, bivariate ETM with the most correlated environmental variable, and bivariate ETM with 4 different prediction methods. The SNP scores were independently drawn from the Bernoulli(0.5) distribution. Polygenic background (adaptive) and residual variance each explained 50% of the phenotypic variance. The genetic correlation between the observed trait and each of the 10 environmental variables defining the environmental gradient was 0.5. (a) Bivariate ETM performed by testing for a common marker effect. (b) Bivariate ETM was performed by testing whether there is any effect on environment or trait.
(PDF)

S6 Fig Power in simulations with a non-adaptive SNP, for single trait mapping (red), bivariate ETM with the most correlated environmental variable (black), and bivariate ETM with 4 different prediction methods (LM, EN, RF, CCA; respectively green, blue, brown and purple). Bivariate ETM was performed by testing for a common marker effect (top) and by testing whether there is any effect on environment or trait (bottom). The causal SNP explained 5% of the variance of the simulated trait, while polygenic background and residual variance explained respectively 45% and 50%. Correlations between true and observed environmental variables were 0.8.
(PDF)

S7 Fig Scatter plots of 61 environmental variables against observed flowering time. Numbers in red indicate squared Pearson correlations.
(PDF)

S8 Fig Scatter plots of 61 environmental variables against predicted flowering time (LM). Numbers in red indicate squared Pearson correlations.
(PDF)

S9 Fig Scatter plots of 61 environmental variables against predicted flowering time (EN). Numbers in red indicate squared Pearson correlations.
(PDF)

S10 Fig Scatter plots of 61 environmental variables against predicted flowering time (RF). Numbers in red indicate squared Pearson correlations.
(PDF)

S11 Fig Scatter plots of 61 environmental variables against predicted flowering time (CCA). Numbers in red indicate squared Pearson correlations.
(PDF)

S12 Fig Recovery results as a function of -log10(p) values. Colors are as in Fig 2. (PDF)

S13 Fig QQ-plot of −log_10_(*p*) values for univariate mapping of the observed trait (first principal component of 3 flowering traits; top) and summer day length (bottom). (PDF)

S14 Fig QQ-plots of −log_10_(*p*) values for univariate mapping of the predicted trait (left column), for bivariate ETM with the test for a common marker effect (middle column) and for bivariate ETM with the test for any marker effect (right column). Four different prediction methods were used (LM, EN, RF, CCA, from top to bottom). (PDF)

S15 Fig QQ-plots of −log_10_(*p*) values for univariate mapping with MLMM (multi-locus mixed model; Segura *et al*. (2012)). The numbers of co-factors selected using the extended BIC criterion were 1,2,0,1,3 and 2, for respectively the observed traits (first row) and for predicted traits (LM and EN (middle row); RF and CCA (bottom row)).
(PDF)

S16 Fig Results comparing enrichment for flowering genes between our method of calling the gene closest to a candidate SNP (shown in blue) and calling all genes within windows of different sizes, 80kb (brown), 40kb (red), 20kb (orange) and 10kb (yellow). The bottom right panel shows results for univariate mapping of the observed trait when calling a maximum of one gene per SNP (i.e. correction for “double hits”).
(PDF)

S1 File List of environmental variables used for trait prediction. (XLS)

S2 File List of flowering genes used in enrichment analysis. (CSV)
