# Supplementary figures and images for: Genome-Wide Association Analysis of Adaptation Using Environmentally Predicted Traits
Source: PLoS Genet. 2015 Oct 23;11(10):e1005594. doi: 10.1371/journal.pgen.1005594 (PMC4619680; doi:10.1371/journal.pgen.1005594)

0.67

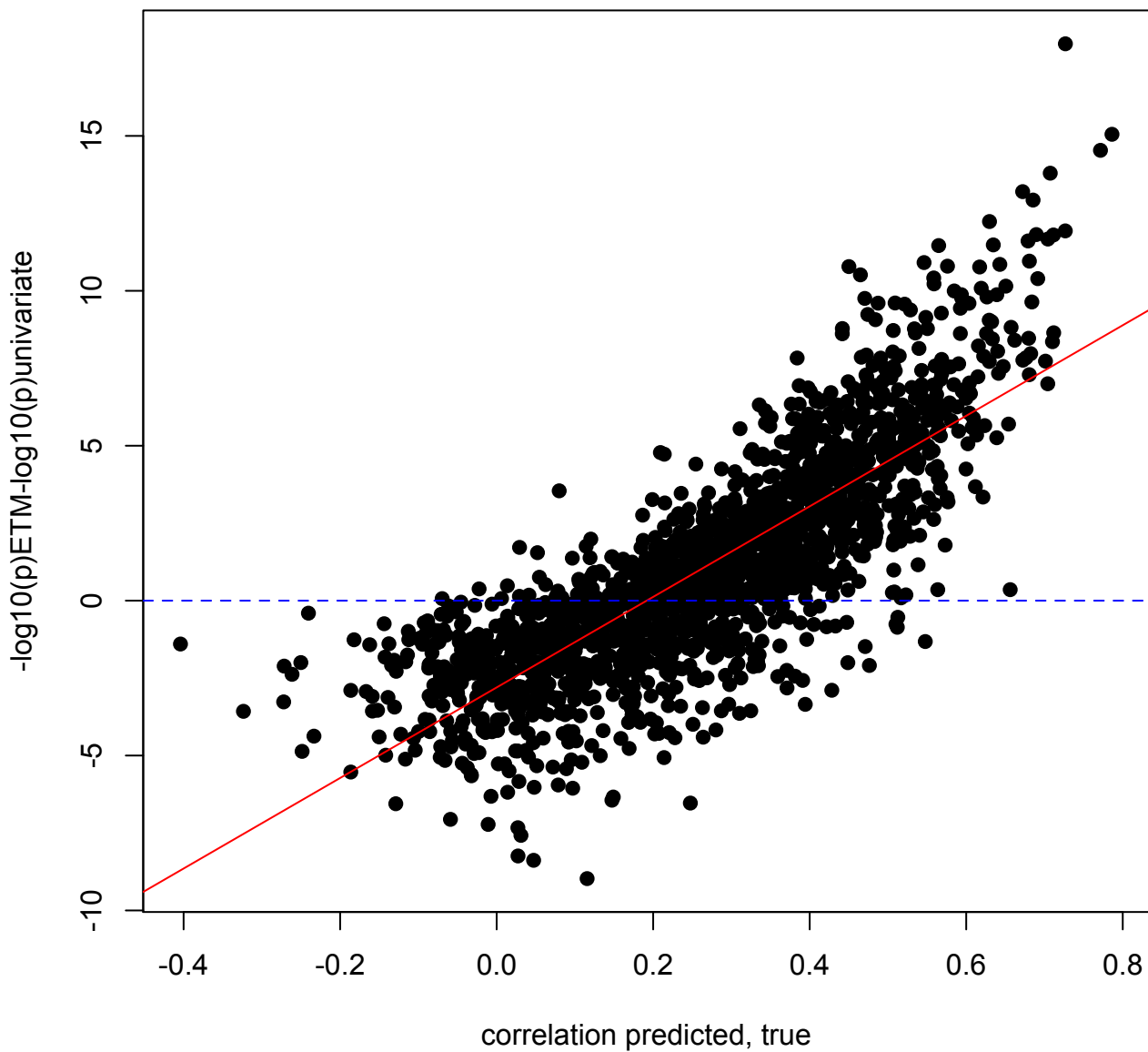

Supplement: S1 Fig — The regression line is shown in red and the line marking equal performance of the two methods is marked in blue. Only results for non-constant predictions are shown. (PDF) [file pgen.1005594.s001.pdf]

A.

Common

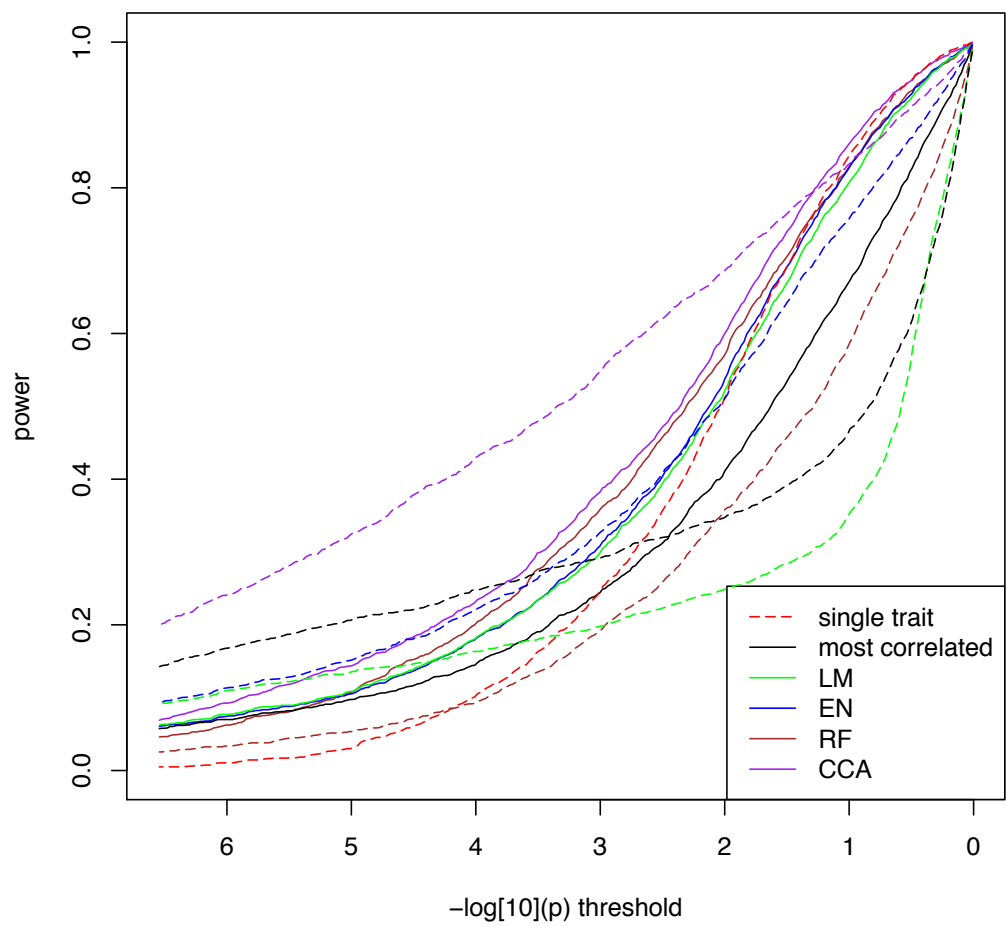

Full

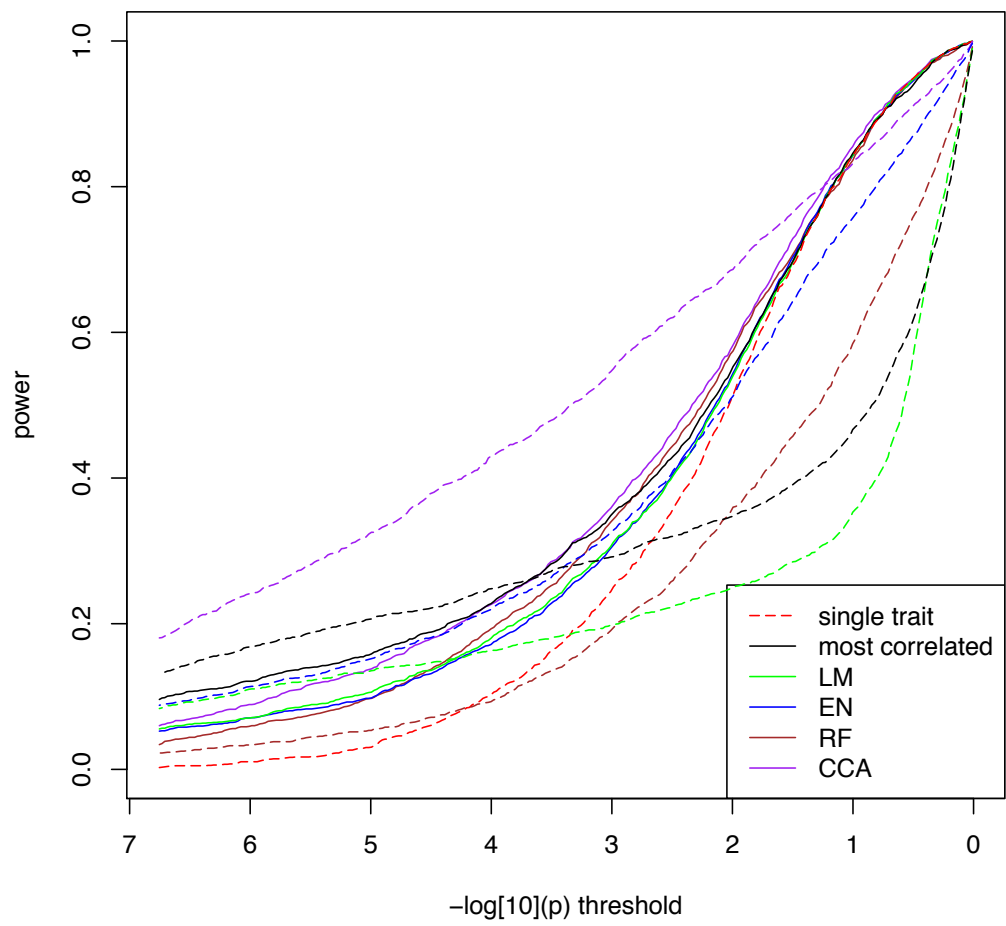

B.

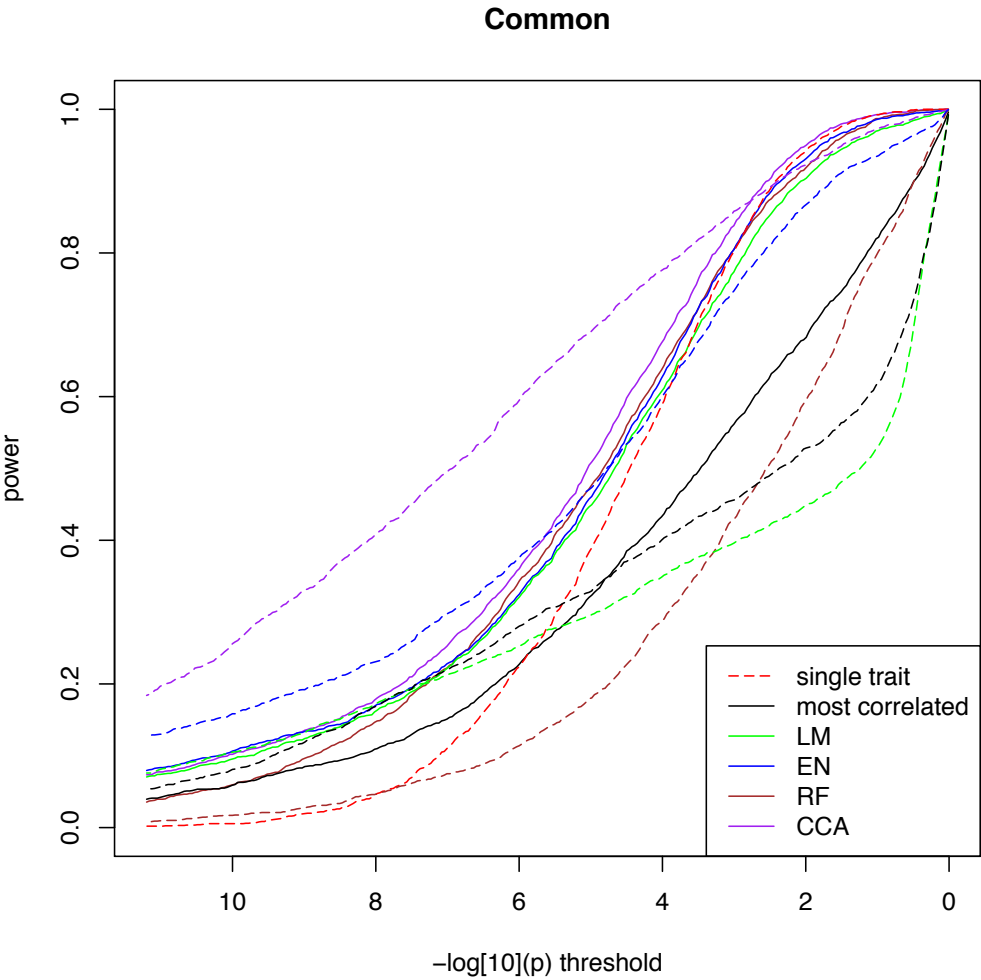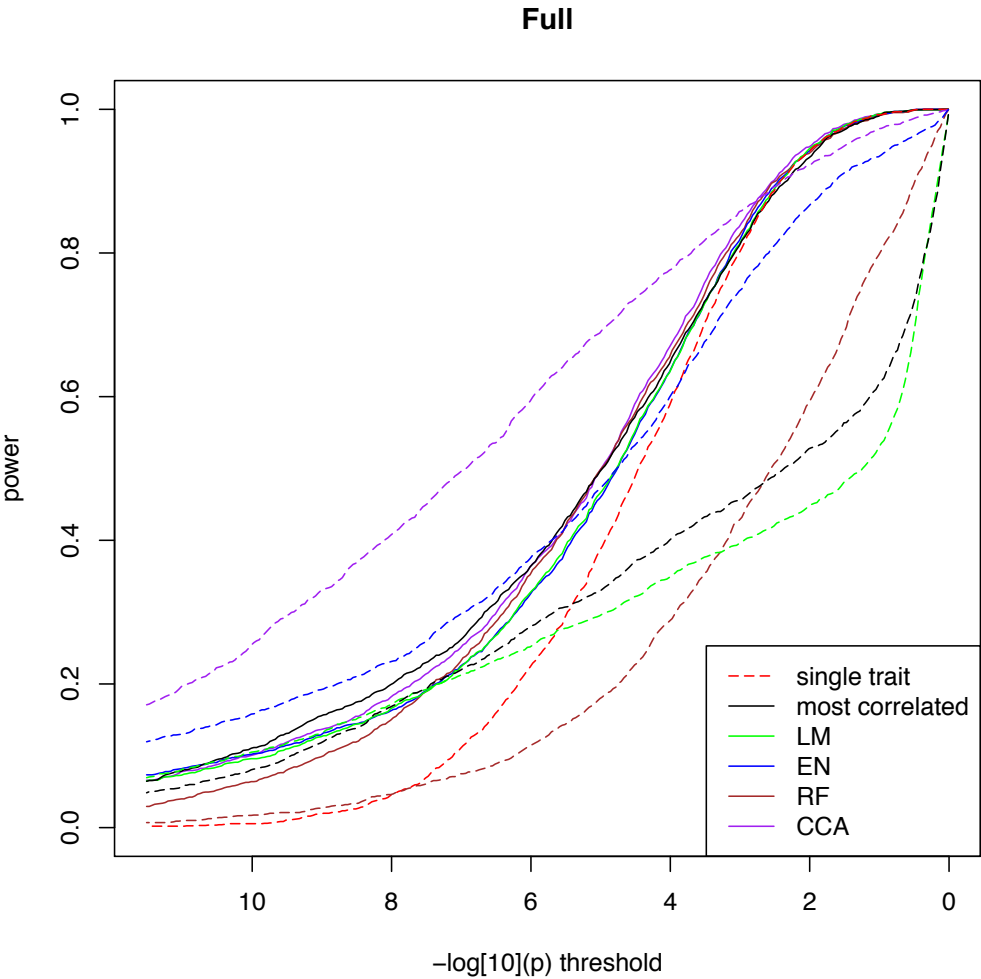

C.

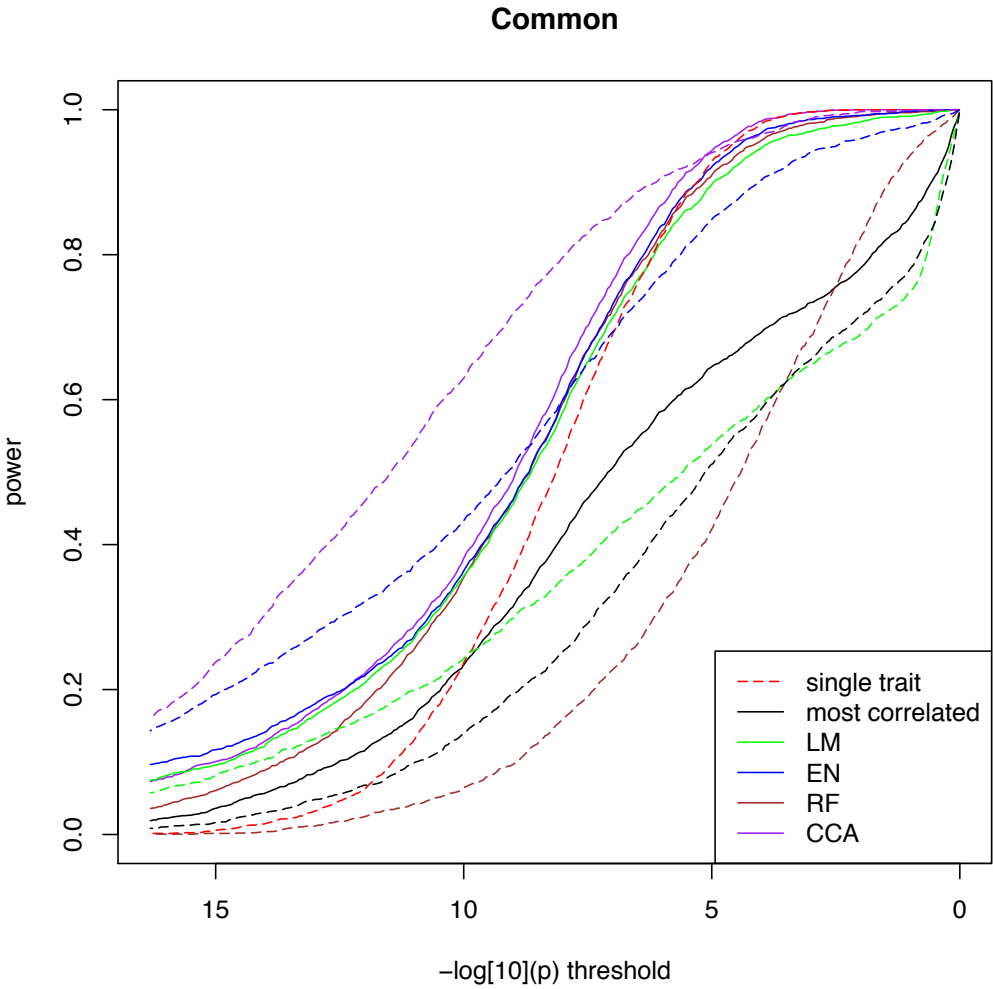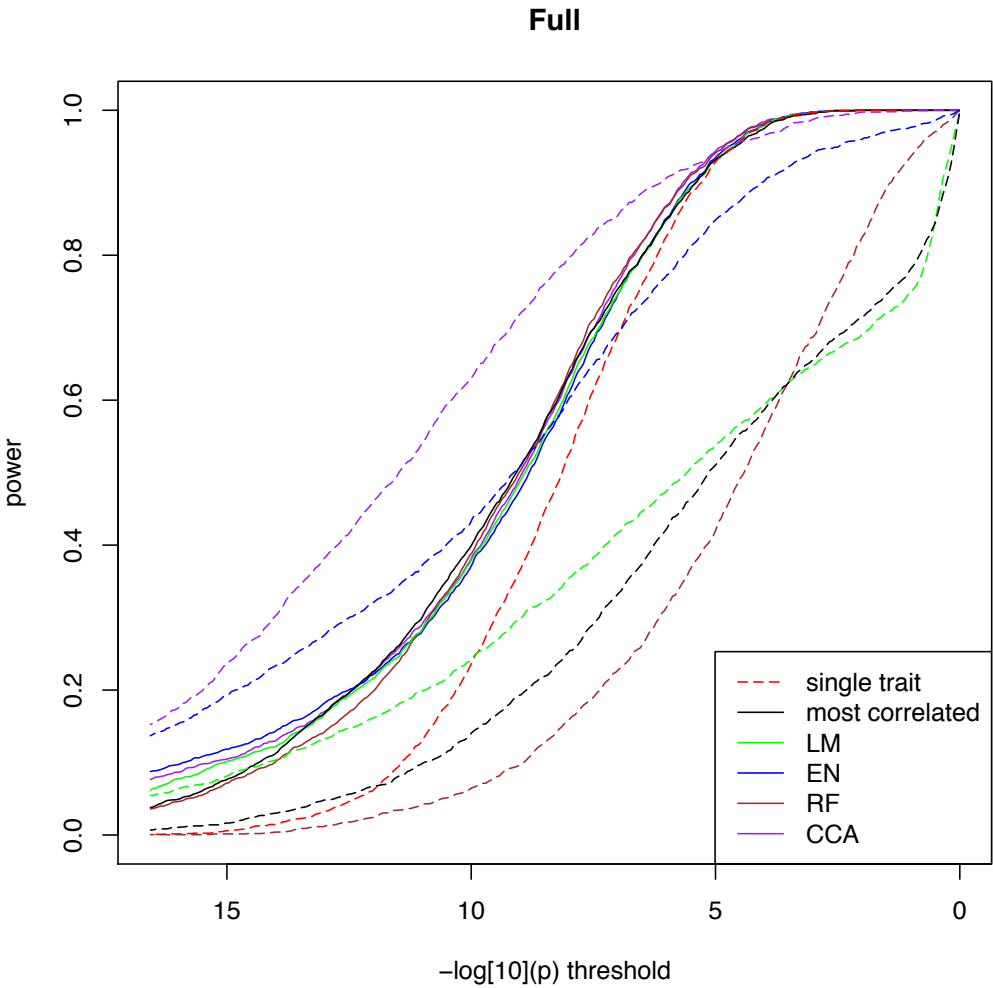

D.

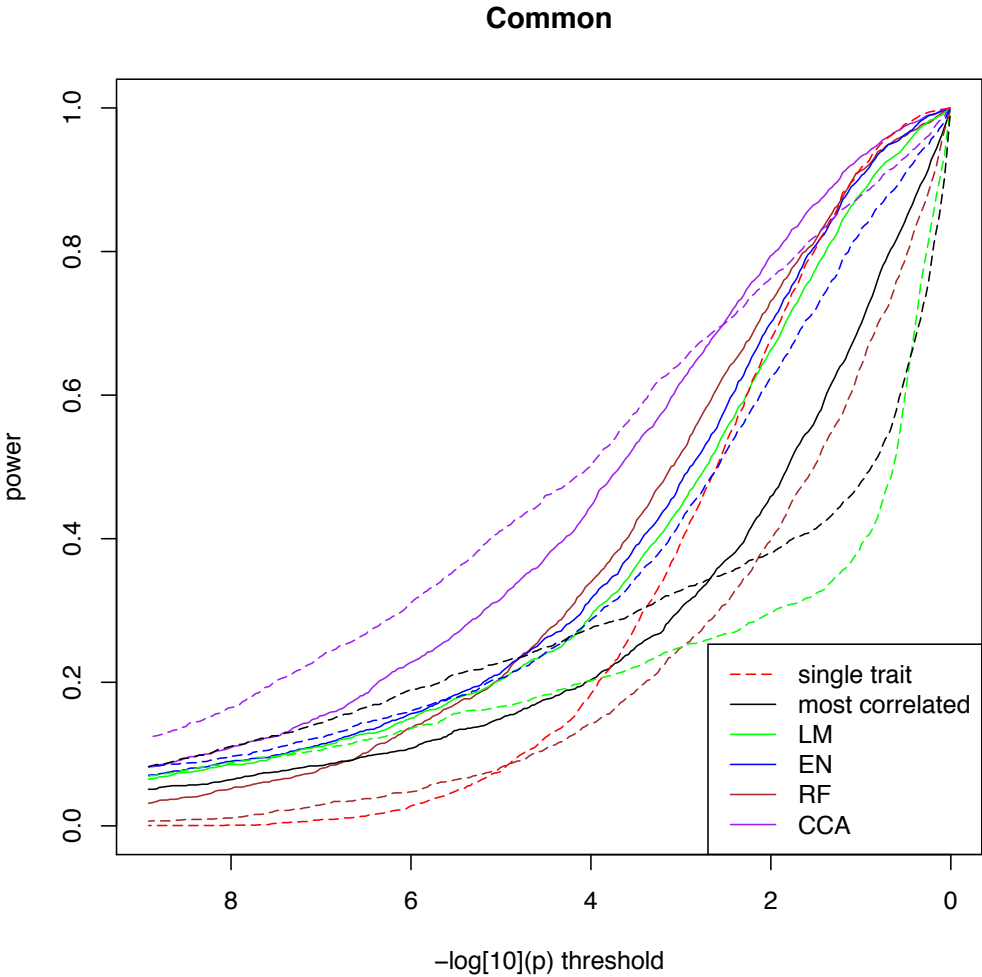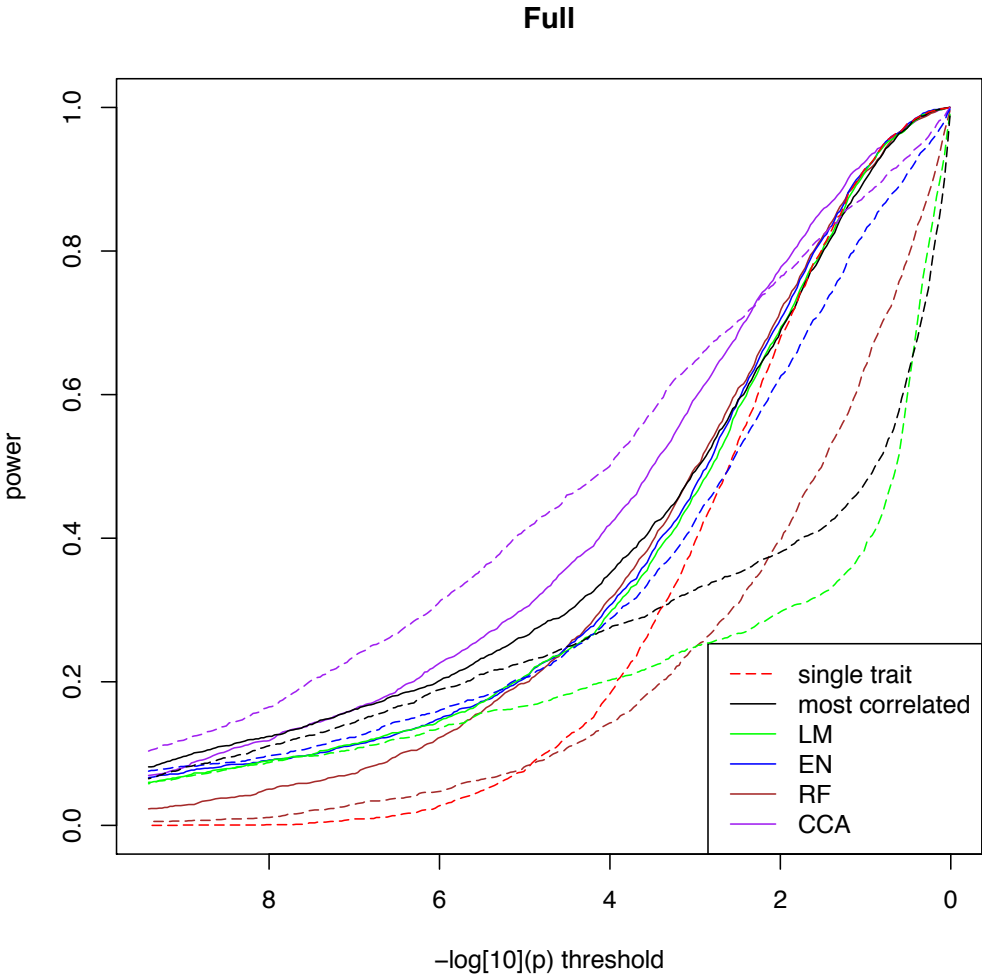

E.

Common

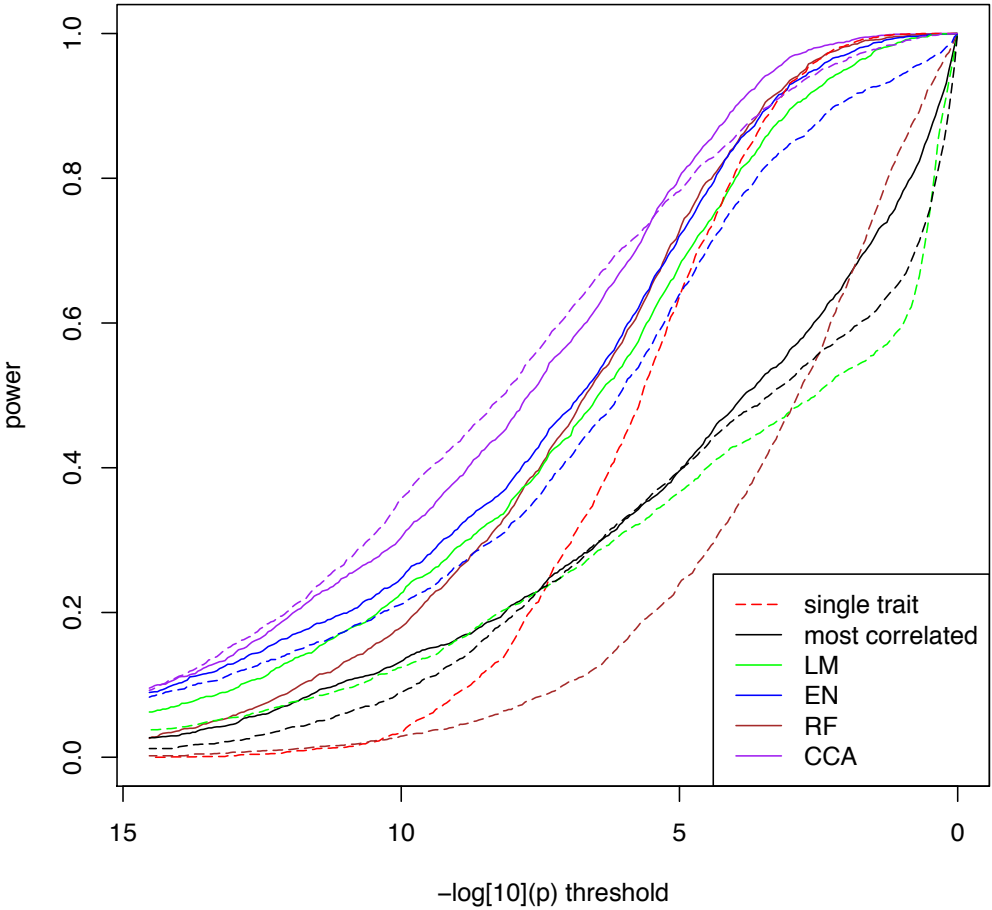

Full

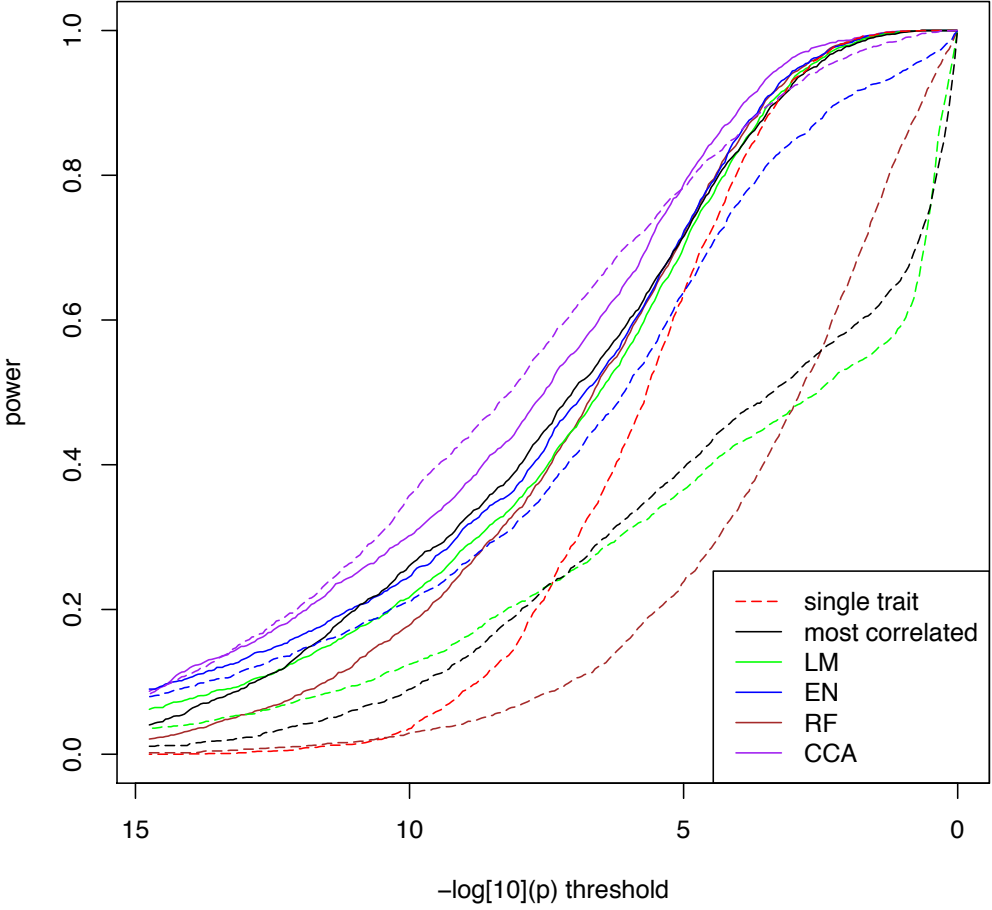

F.

Common

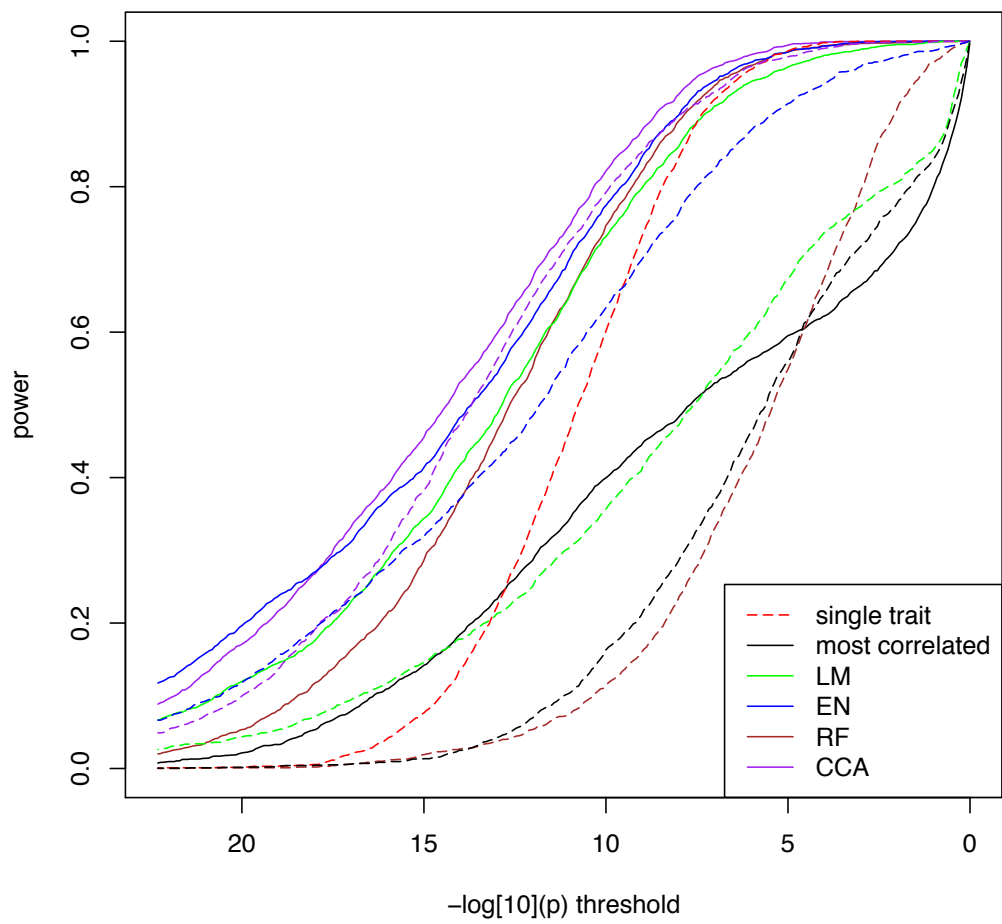

Full

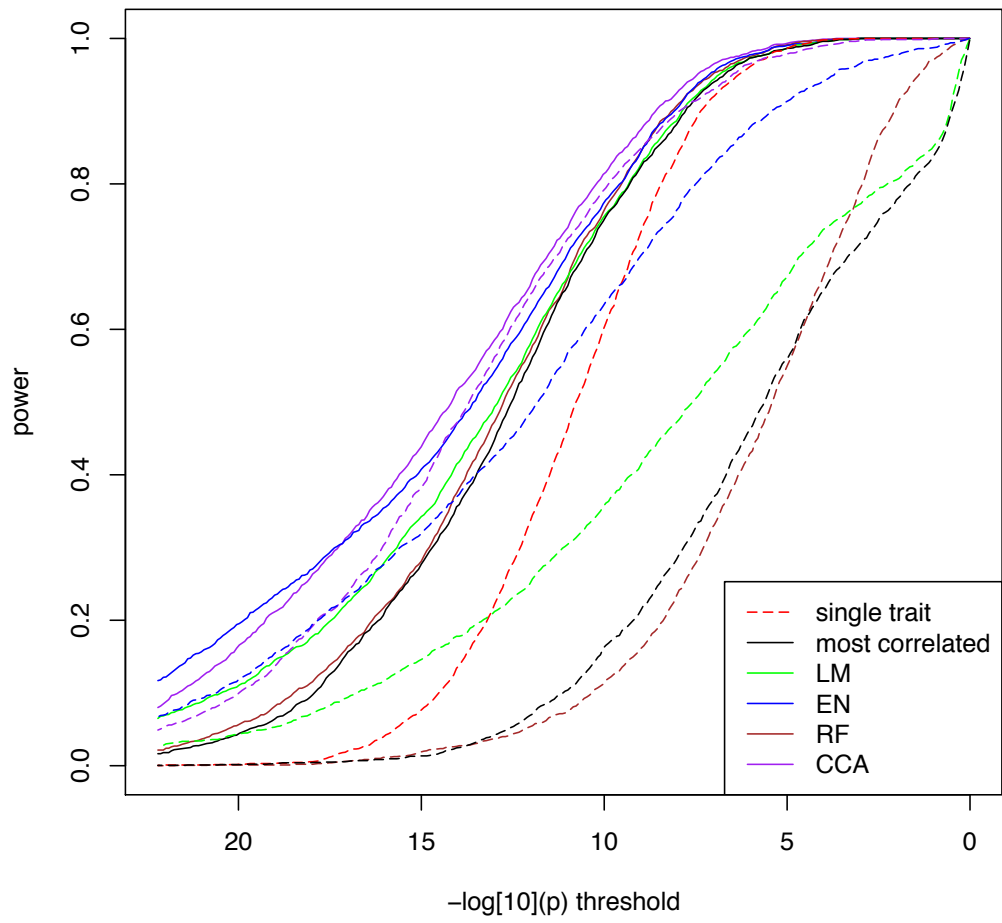

G.

Common

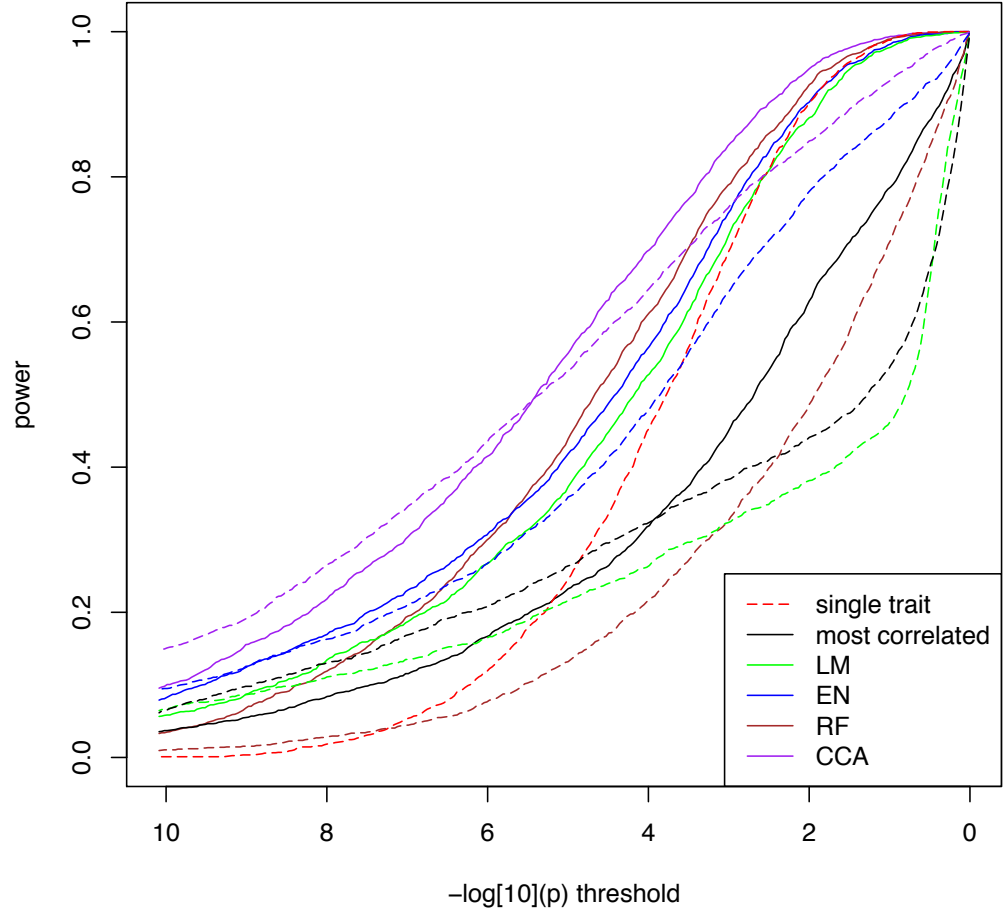

Full

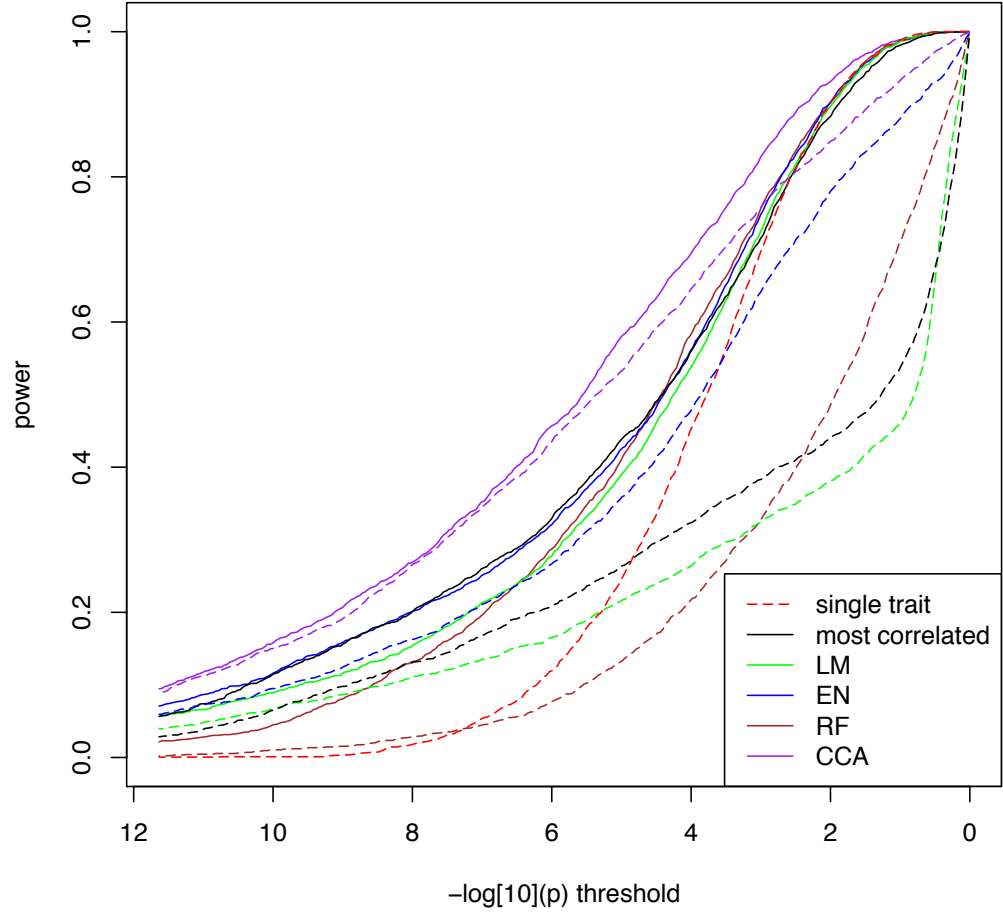

H.

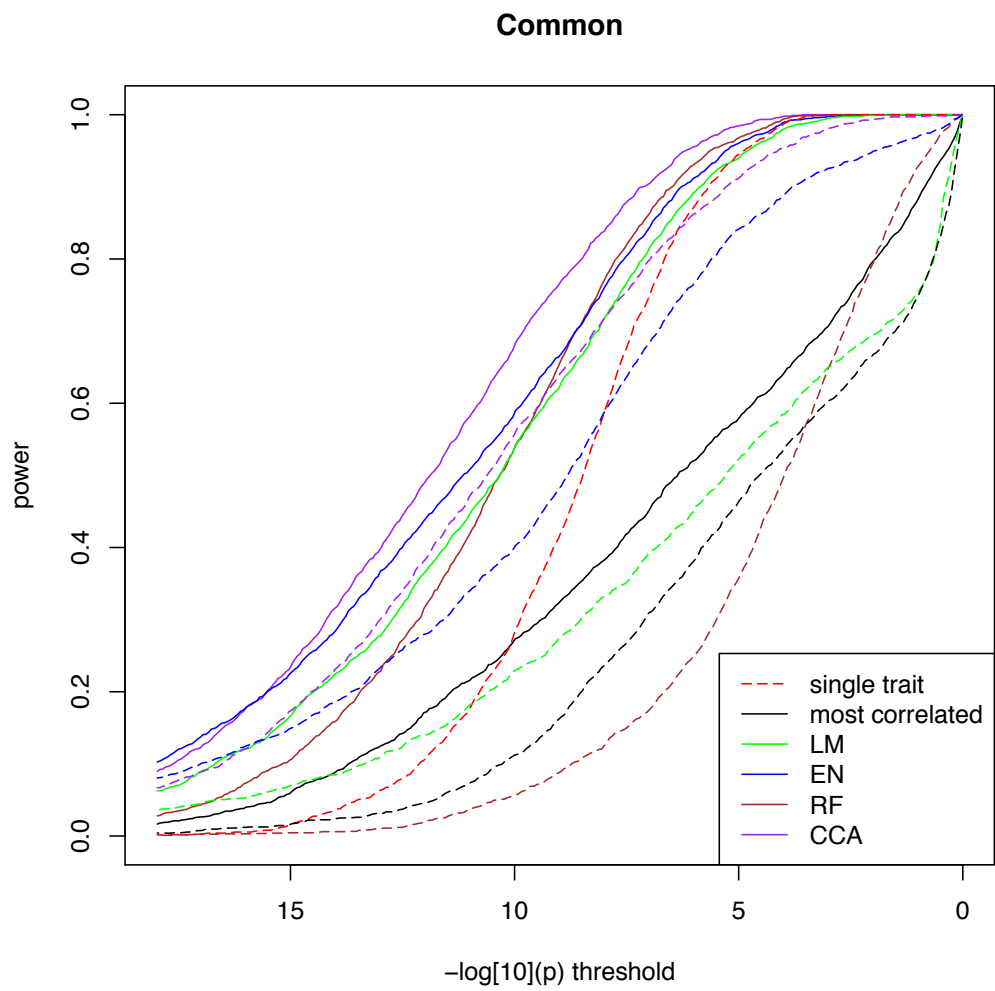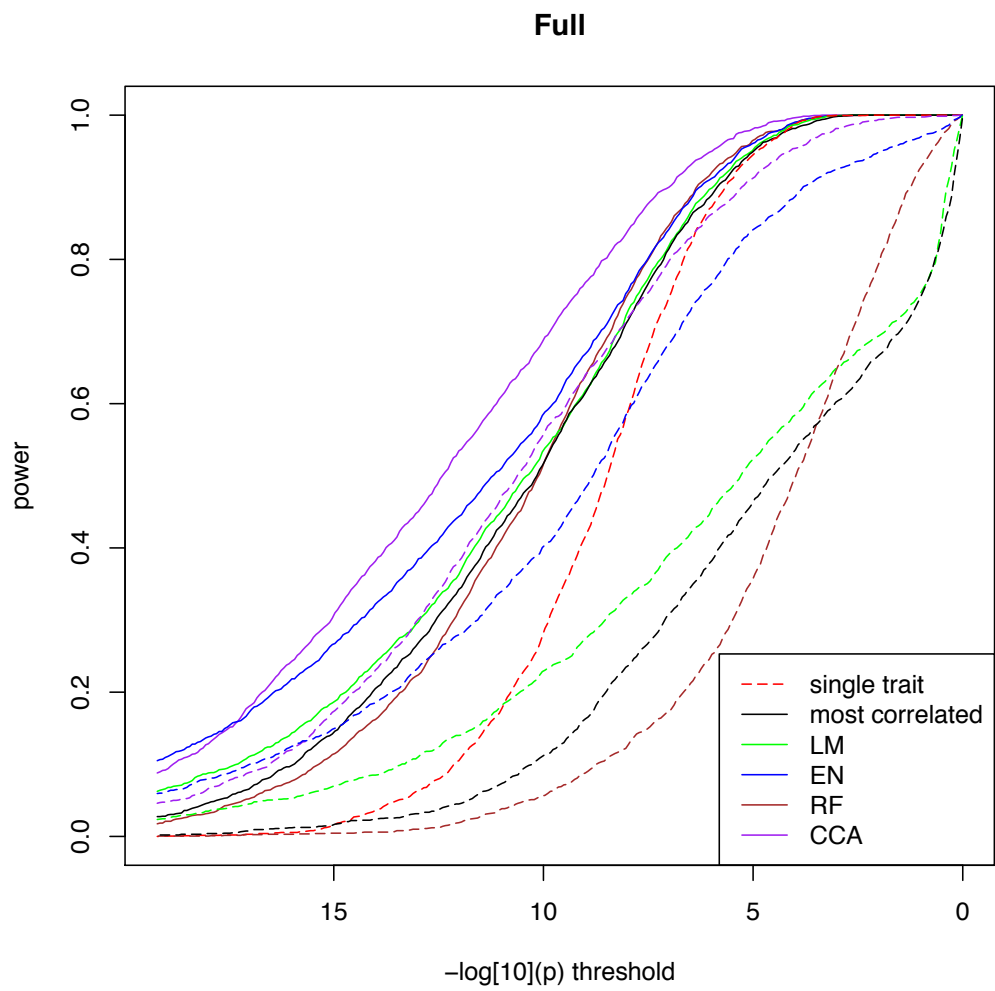

I.

### Common

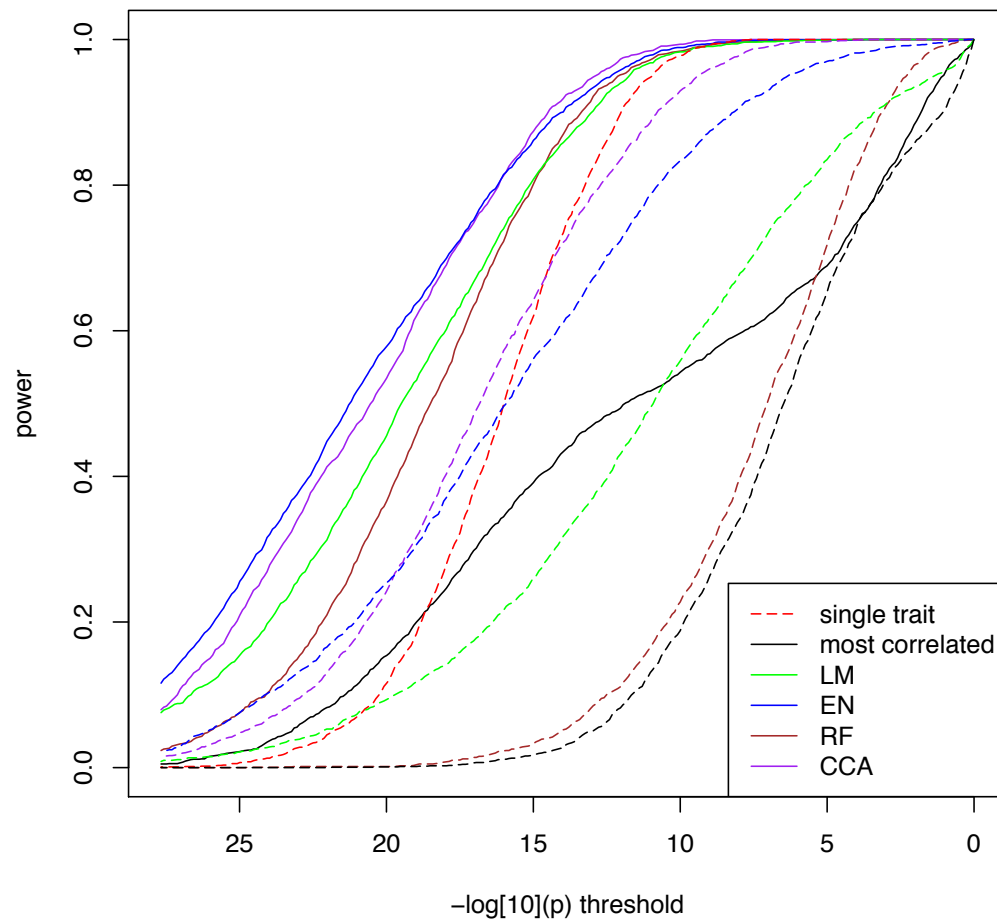

### Full

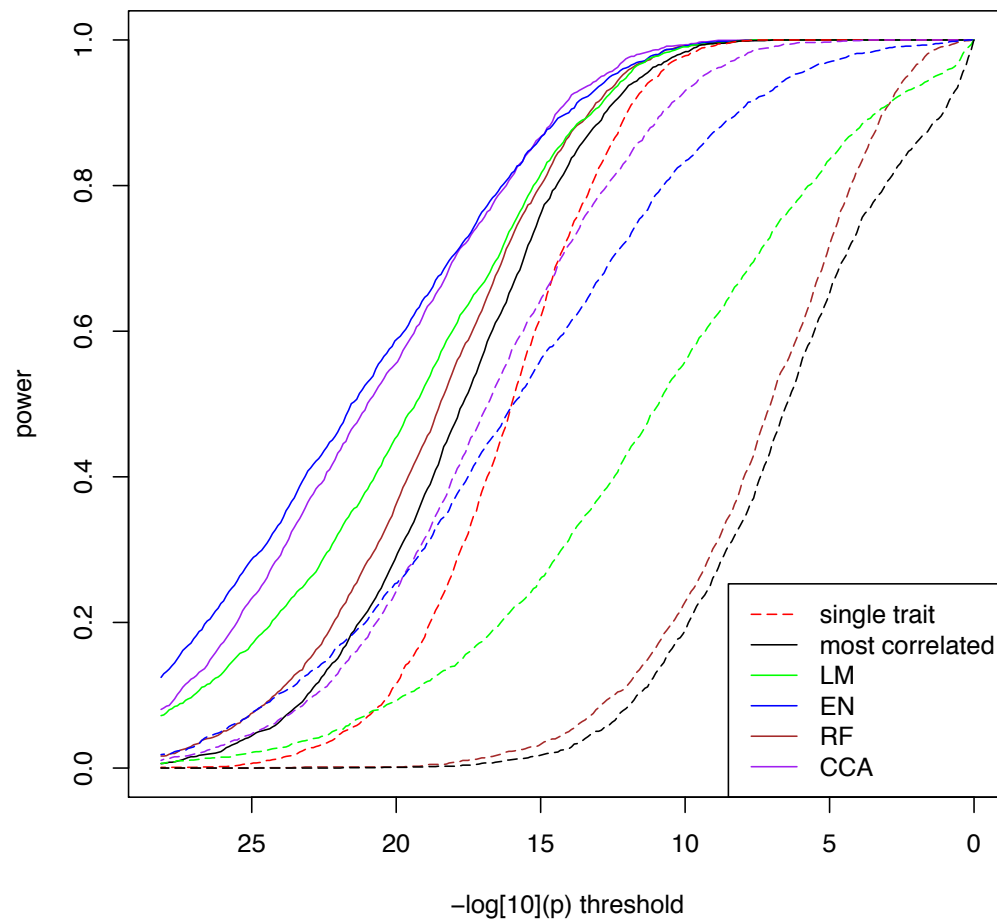

Supplement: S2 Fig — Bivariate ETM was performed by testing for a common marker effect (top) and by testing whether there is any effect on environment or trait (bottom). (a) h 2 = 0.2. The causal SNP explained 2% of the variance of the simulated trait, while polygenic background and residual variance explained respectively 18% and 80%. Correlations between true and observed environmental variables were 0.8. (b) h 2 = 0.2. The causal SNP explained 5% of the variance of the simulated trait, while polygenic background and residual variance explained respectively 15% and 80%. Correlations between true and observed environmental variables were 0.8. (c) h 2 = 0.2. The causal SNP explained 10% of the variance of the simulated trait, while polygenic background and residual variance explained respectively 10% and 80%. Correlations between true and observed environmental variables were 0.8. (d) h 2 = 0.5. The causal SNP explained 2% of the variance of the simulated trait, while polygenic background and residual variance explained respectively 48% and 50%. Correlations between true and observed environmental variables were 0.8. (e) h 2 = 0.5. The causal SNP explained 5% of the variance of the simulated trait, while polygenic background and residual variance explained respectively 45% and 50%. Correlations between true and observed environmental variables were 0.8. (f) h 2 = 0.5. The causal SNP explained 10% of the variance of the simulated trait, while polygenic background and residual variance explained respectively 40% and 50%. Correlations between true and observed environmental variables were 0.8. (g) h 2 = 0.8. The causal SNP explained 2% of the variance of the simulated trait, while polygenic background and residual variance explained respectively 78% and 20%. Correlations between true and observed environmental variables were 0.8. (h) h 2 = 0.8. The causal SNP explained 5% of the variance of the simulated trait, while polygenic background and residual variance explained respectively 75% and [file pgen.1005594.s002.pdf]

### Common

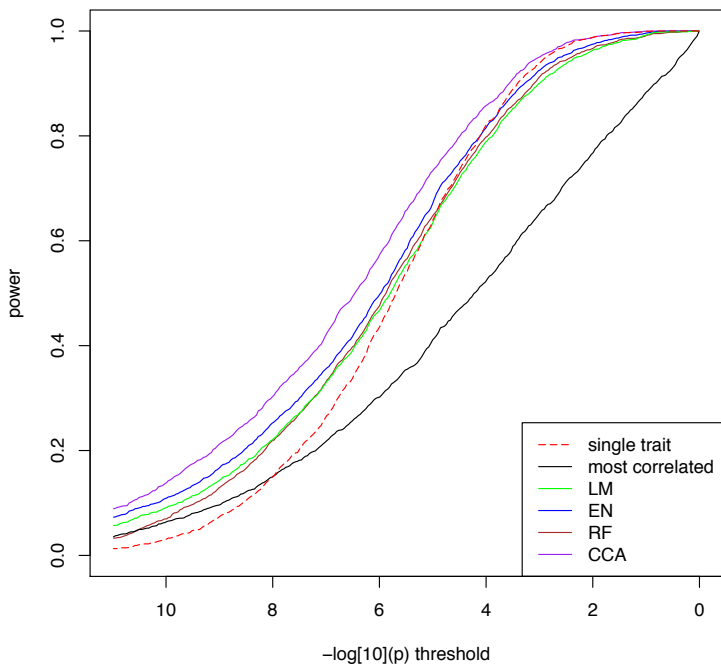

### Full

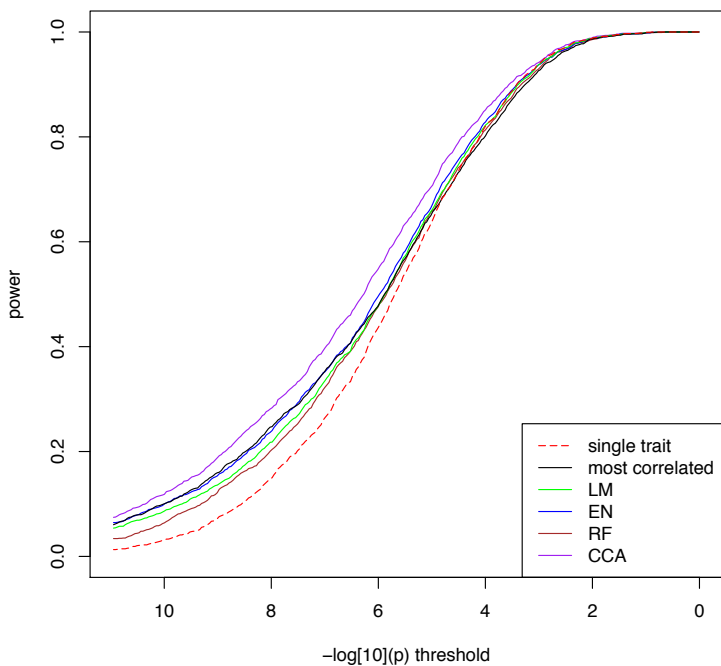

Supplement: S3 Fig — Bivariate ETM was performed by testing for a common marker effect (top) and by testing whether there is any effect on environment or trait (bottom). The causal SNP explained 45% of the variance of the simulated trait, while polygenic background and residual variance explained respectively 45% and 50%. (PDF) [file pgen.1005594.s003.pdf]

### Common

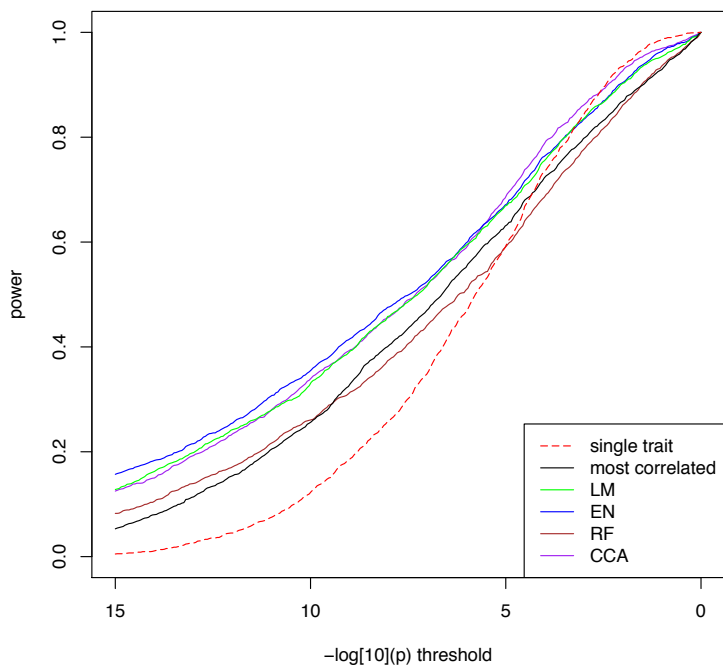

### Full

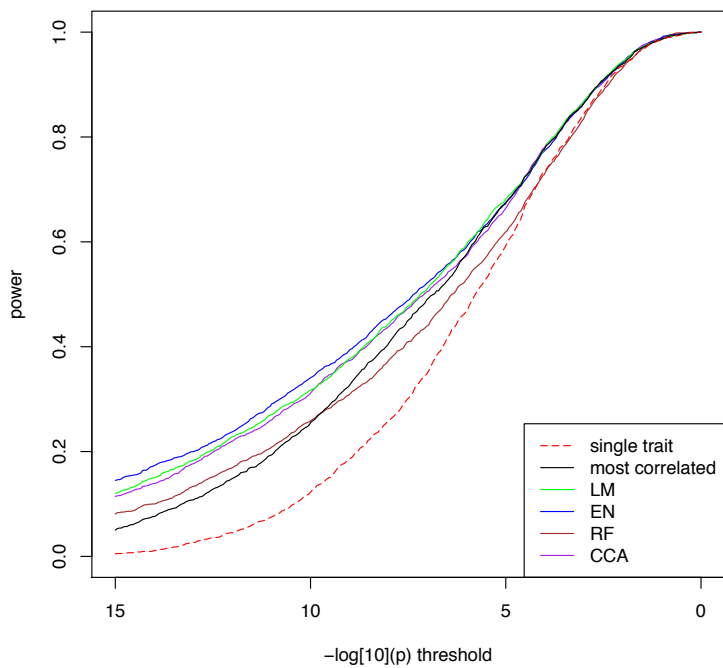

Supplement: S4 Fig — Bivariate ETM was performed by testing for a common marker effect (top) and by testing whether there is any effect on environment or trait (bottom). The causal SNP explained 5% of the variance of the simulated trait, while polygenic background and residual variance explained respectively 45% and 50%. (PDF) [file pgen.1005594.s004.pdf]

**single trait**

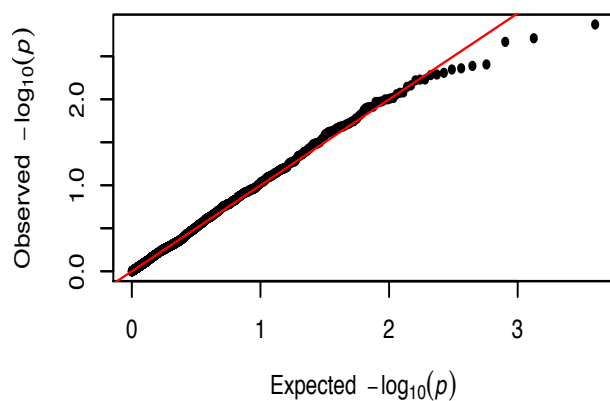

**most correlated**

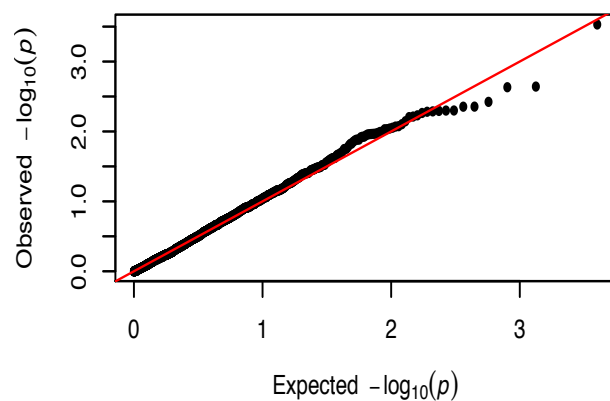

**LM**

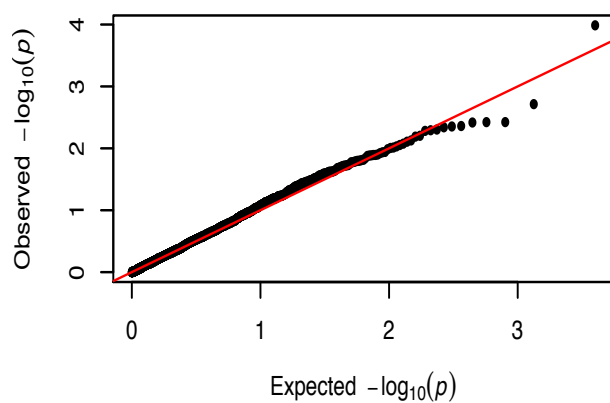

**EN**

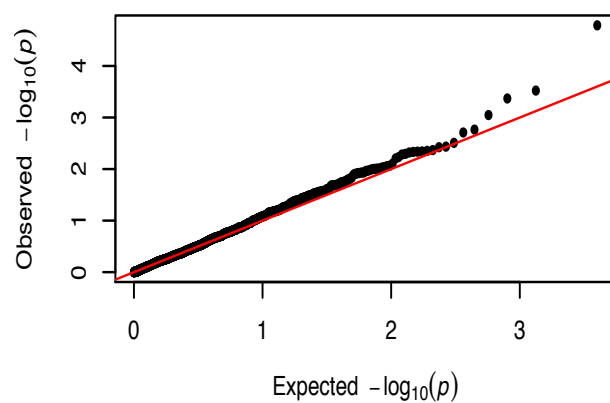

**RF**

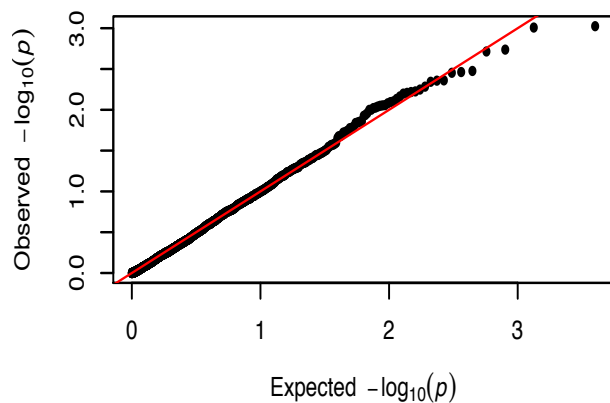

**CCA**

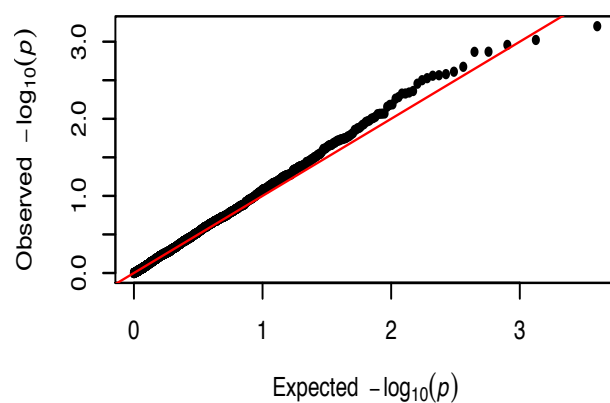

single trait

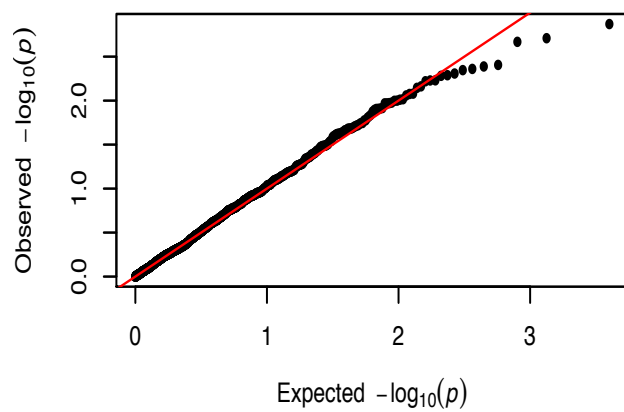

most correlated

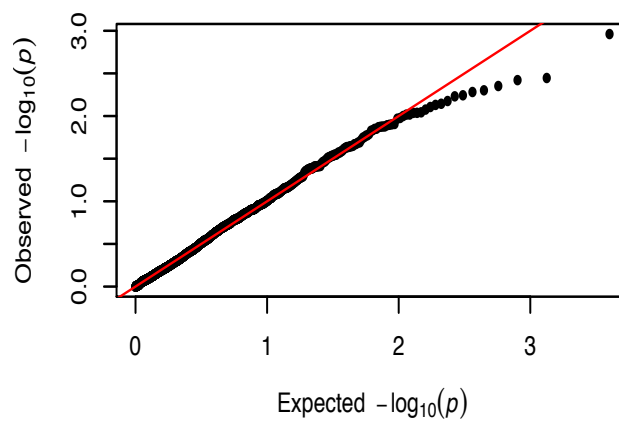

LM

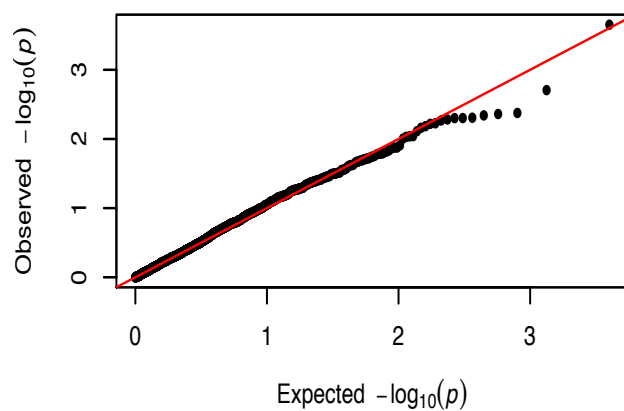

EN

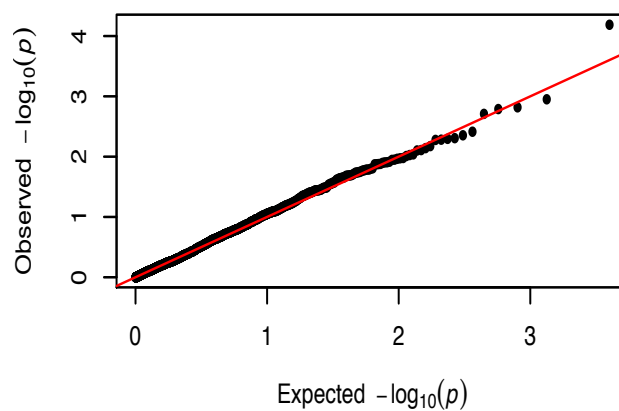

RF

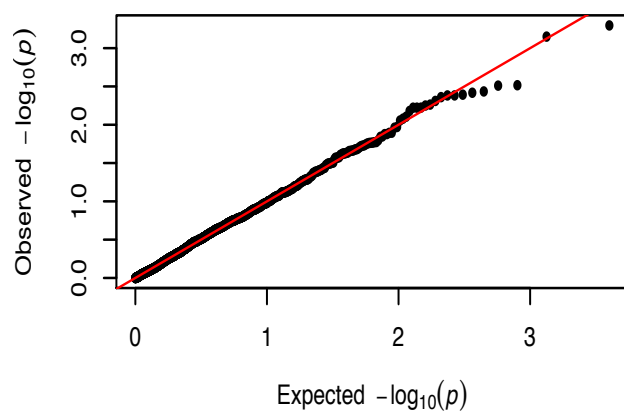

CCA

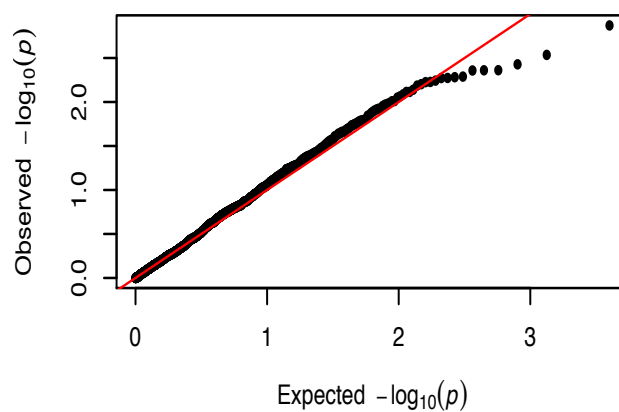

Supplement: S5 Fig — The SNP scores were independently drawn from the Bernoulli(0.5) distribution. Polygenic background (adaptive) and residual variance each explained 50% of the phenotypic variance. The genetic correlation between the observed trait and each of the 10 environmental variables defining the environmental gradient was 0.5. (a) Bivariate ETM performed by testing for a common marker effect. (b) Bivariate ETM was performed by testing whether there is any effect on environment or trait. (PDF) [file pgen.1005594.s005.pdf]

### Common

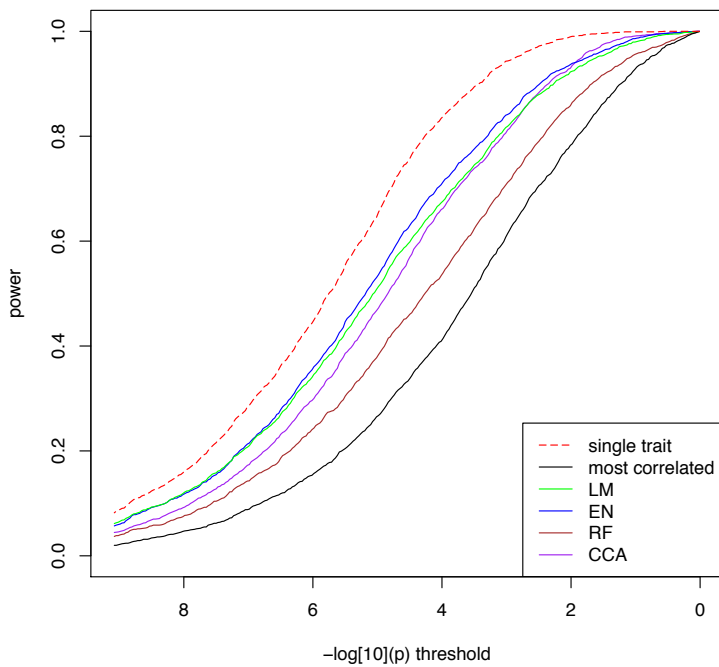

### Full

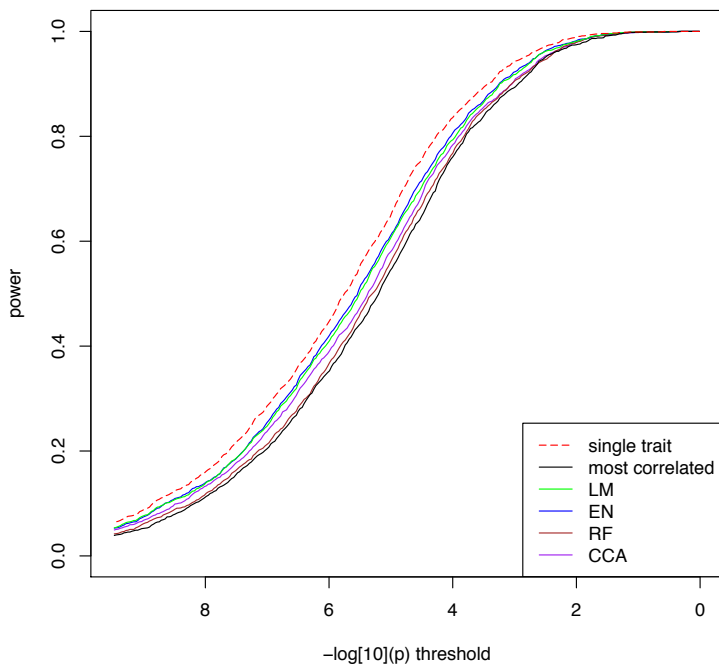

Supplement: S6 Fig — Bivariate ETM was performed by testing for a common marker effect (top) and by testing whether there is any effect on environment or trait (bottom). The causal SNP explained 5% of the variance of the simulated trait, while polygenic background and residual variance explained respectively 45% and 50%. Correlations between true and observed environmental variables were 0.8. (PDF) [file pgen.1005594.s006.pdf]

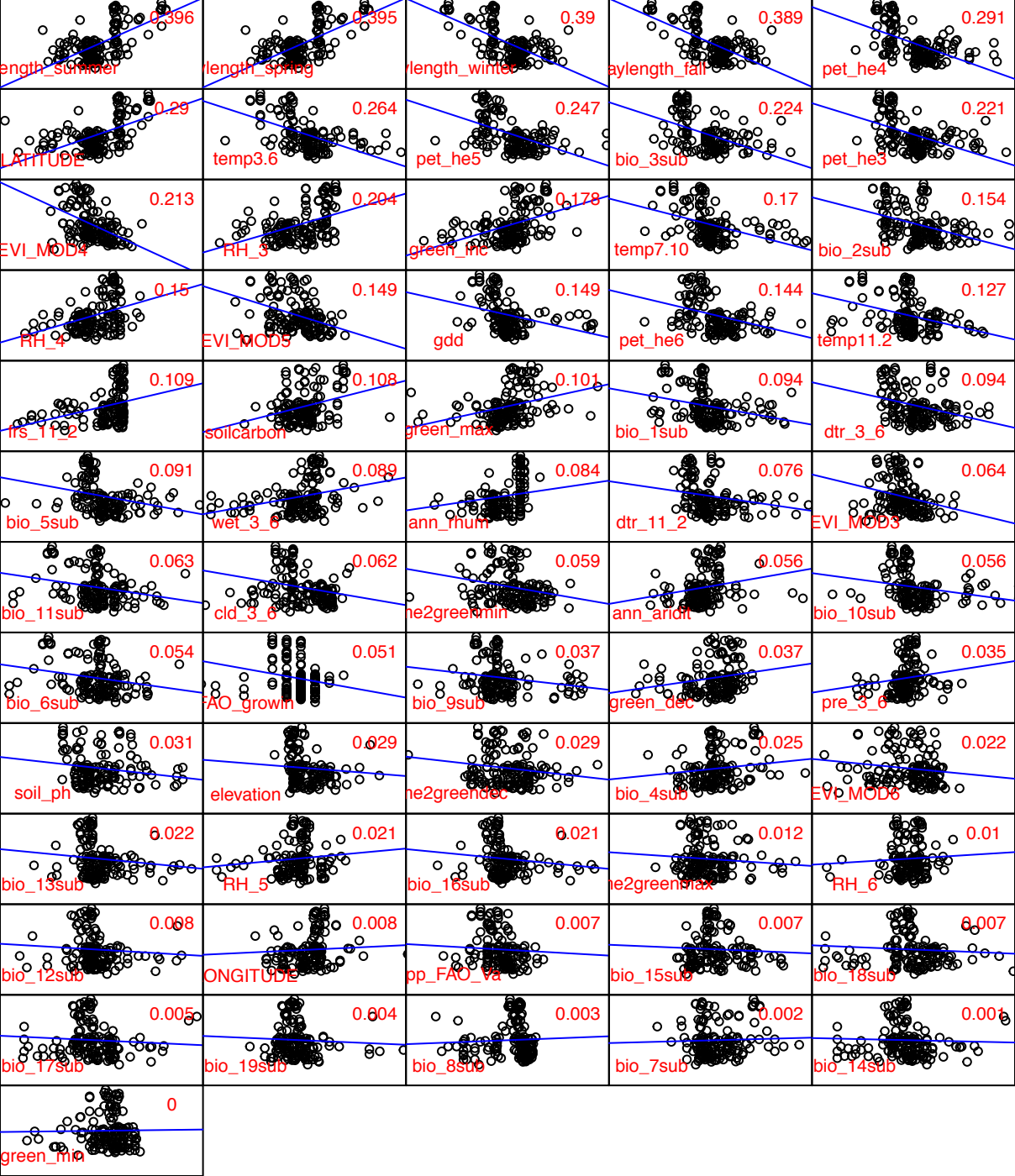

Supplement: S7 Fig — Numbers in red indicate squared Pearson correlations. (PDF) [file pgen.1005594.s007.pdf]

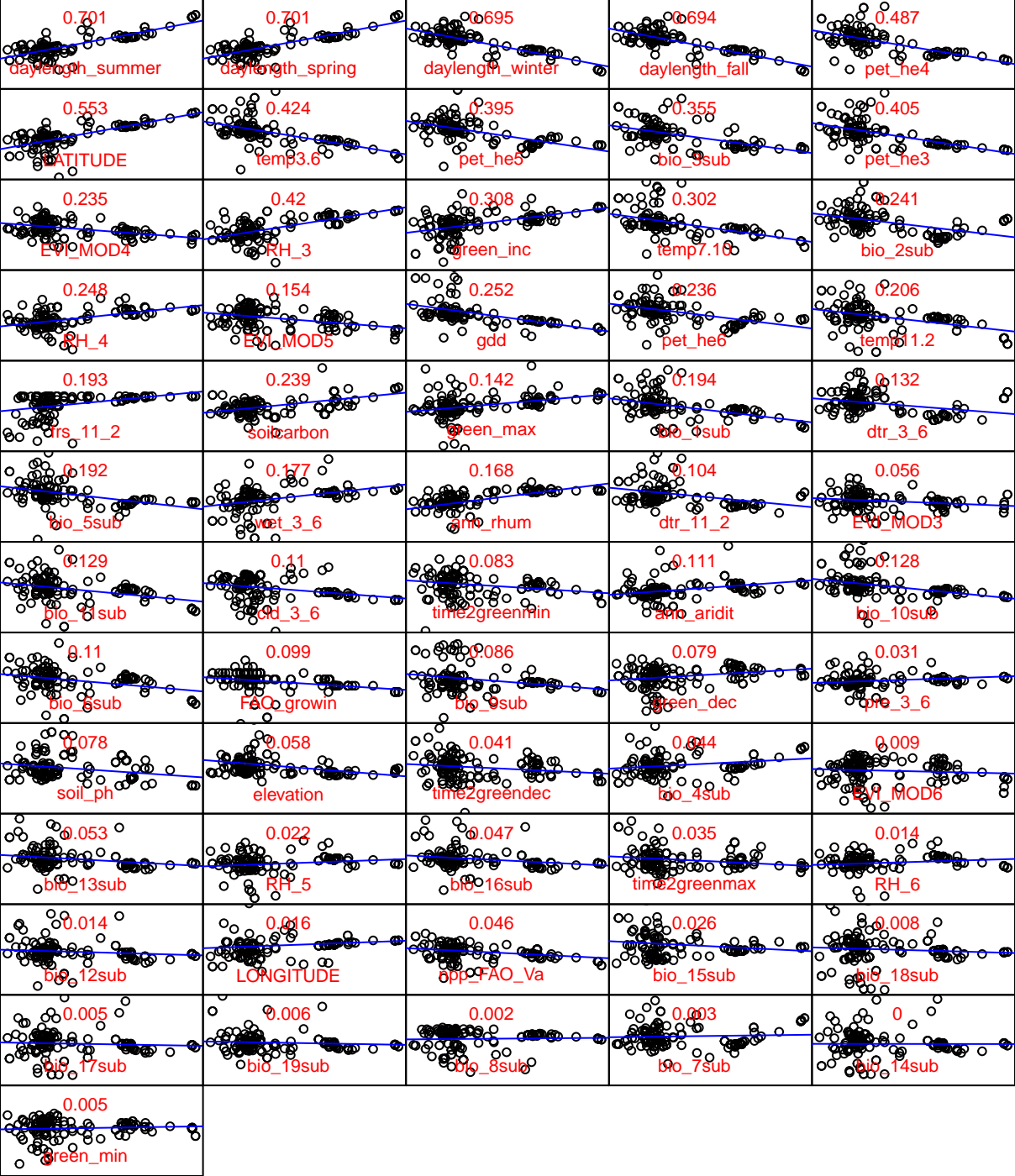

Supplement: S8 Fig — Numbers in red indicate squared Pearson correlations. (PDF) [file pgen.1005594.s008.pdf]

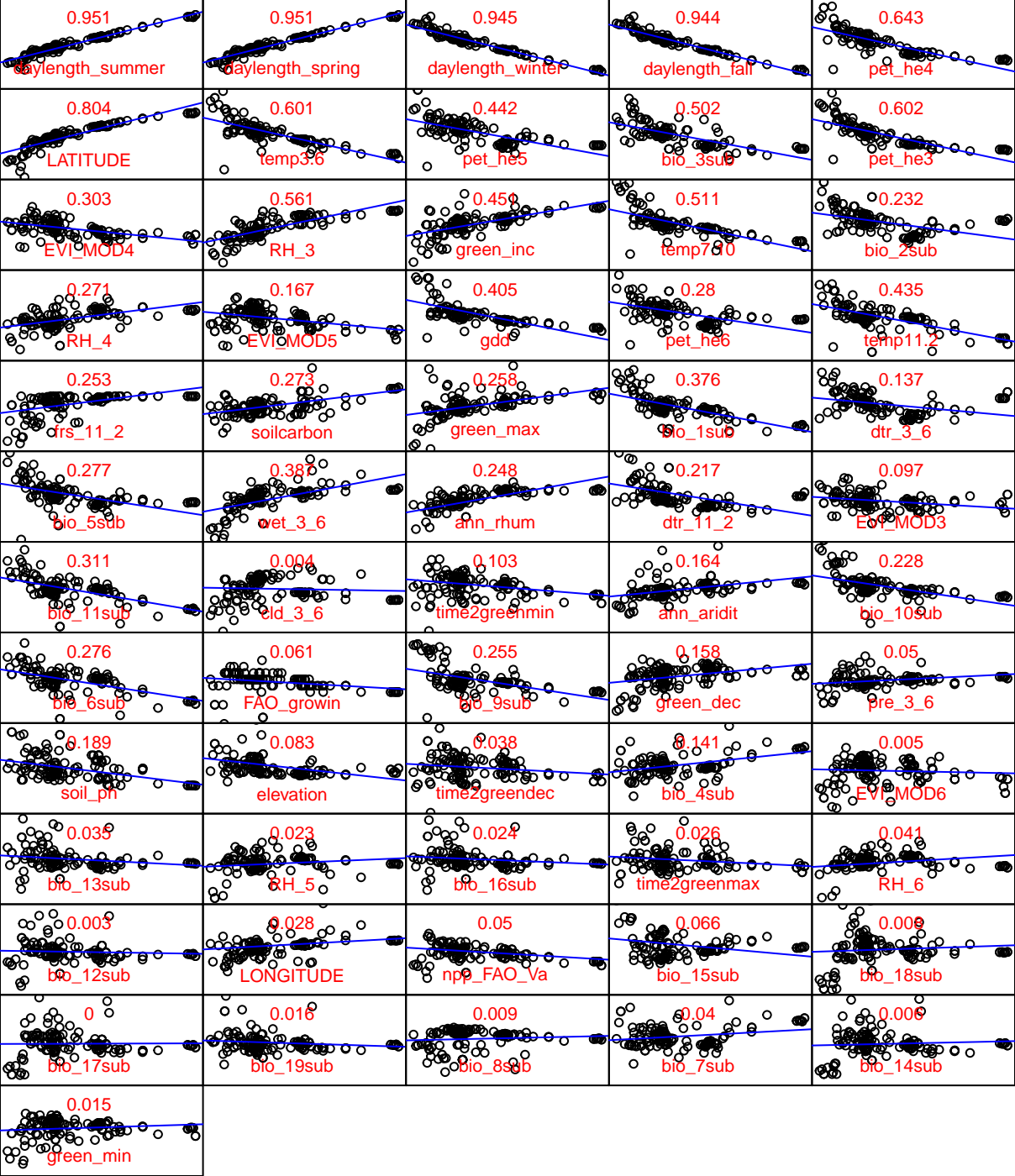

Supplement: S9 Fig — Numbers in red indicate squared Pearson correlations. (PDF) [file pgen.1005594.s009.pdf]

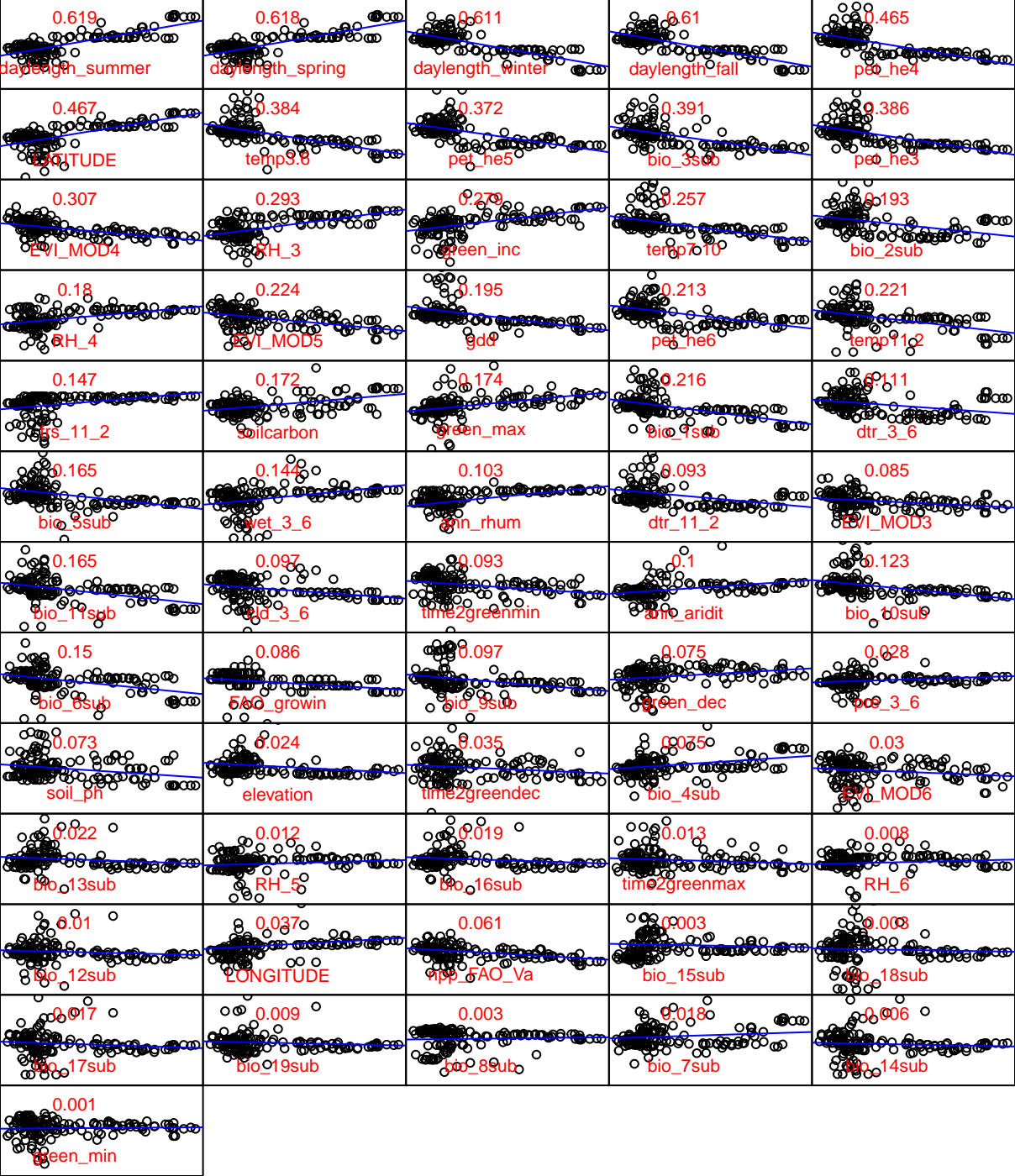

Supplement: S10 Fig — Numbers in red indicate squared Pearson correlations. (PDF) [file pgen.1005594.s010.pdf]

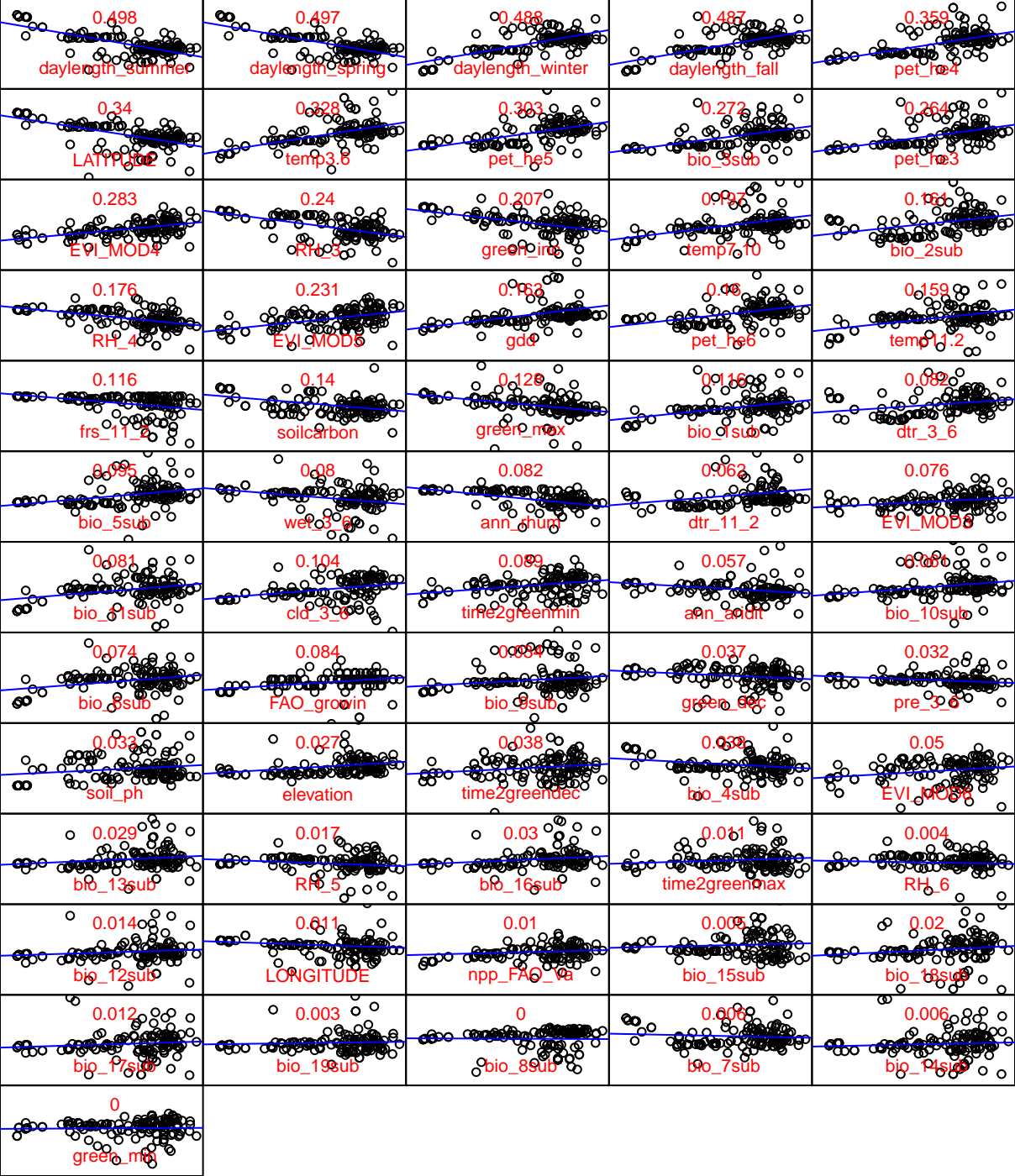

Supplement: S11 Fig — Numbers in red indicate squared Pearson correlations. (PDF) [file pgen.1005594.s011.pdf]

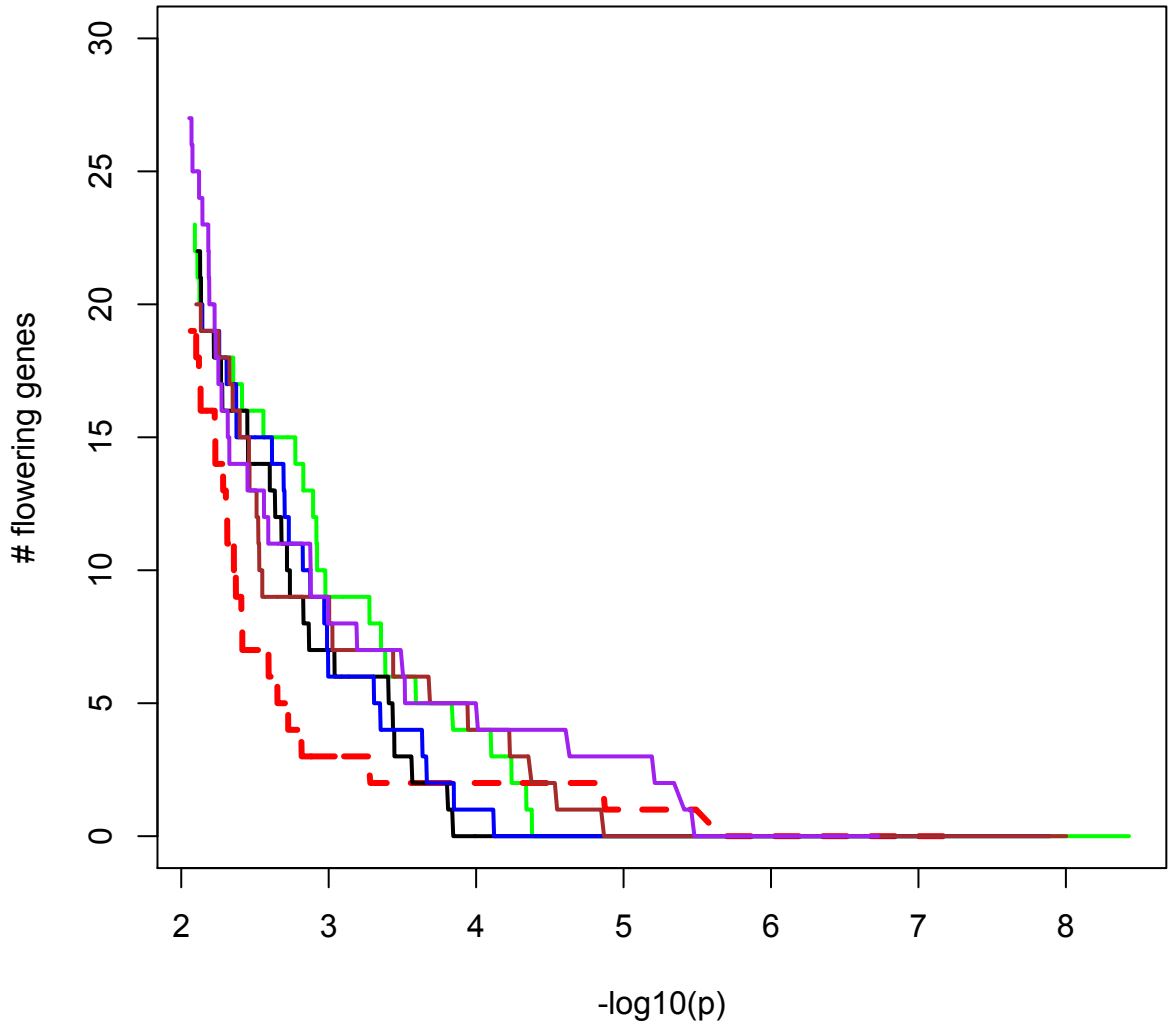

Supplement: S12 Fig — (PDF) [file pgen.1005594.s012.pdf]

trait

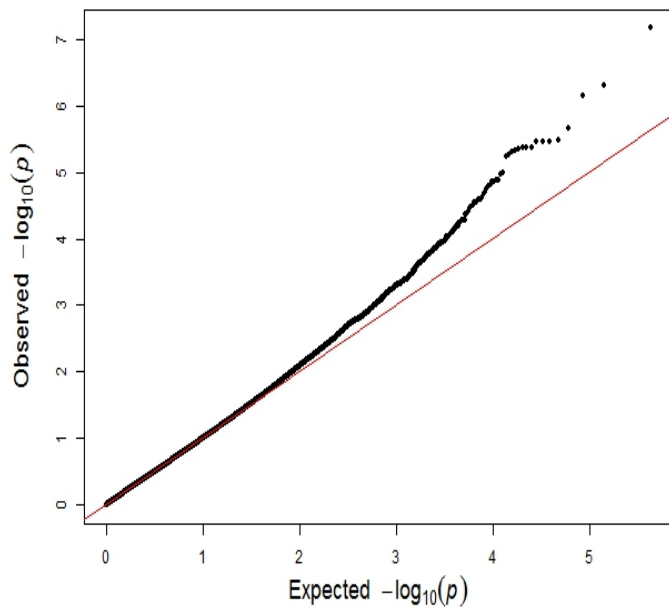

daylength\_summer

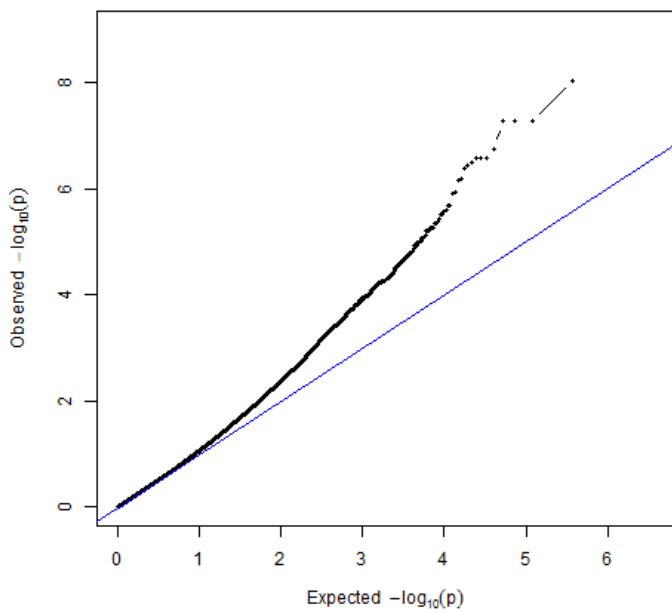

Supplement: S13 Fig — (PDF) [file pgen.1005594.s013.pdf]

trait\_obs

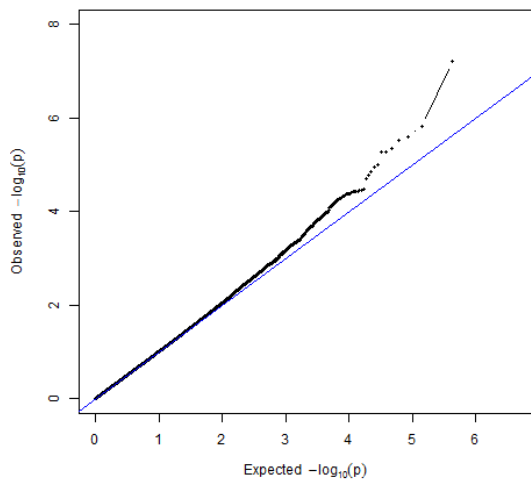

CCA\_obs

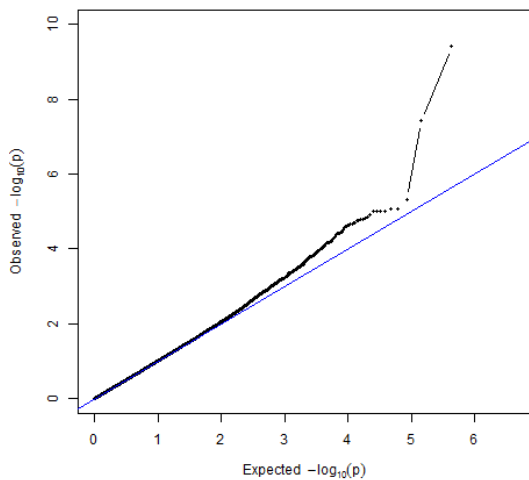

LM\_pred

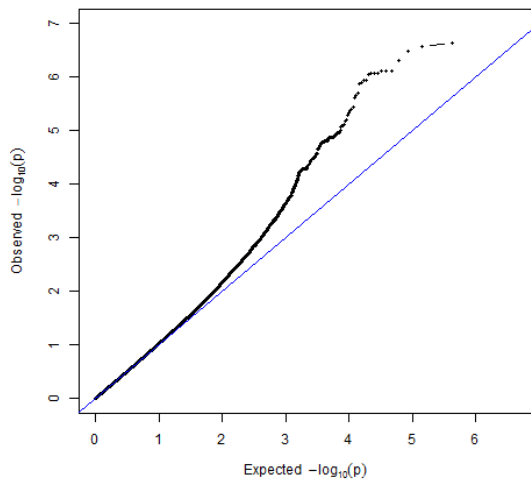

EN\_pred

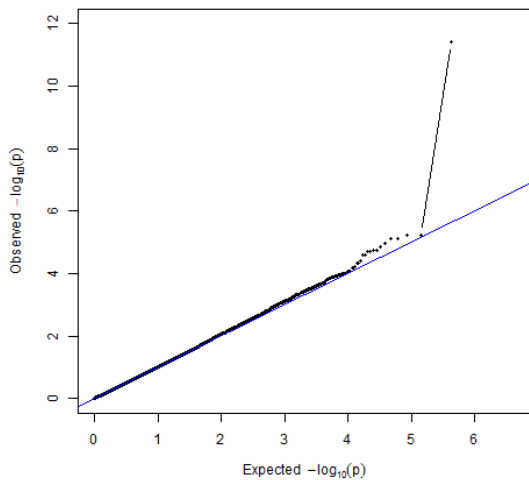

RF\_pred

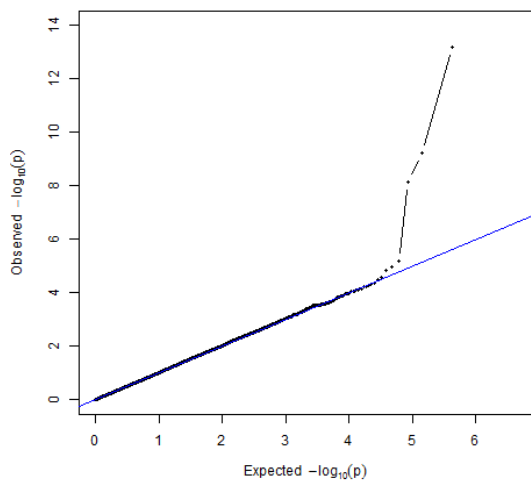

CCA\_pred

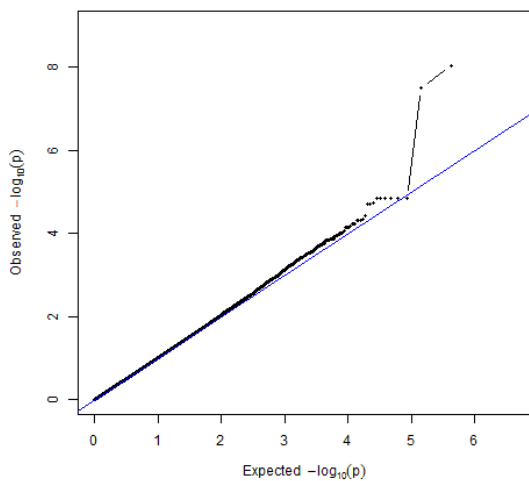

Supplement: S15 Fig — The numbers of co-factors selected using the extended BIC criterion were 1,2,0,1,3 and 2, for respectively the observed traits (first row) and for predicted traits (LM and EN (middle row); RF and CCA (bottom row)). (PDF) [file pgen.1005594.s015.pdf]

lm

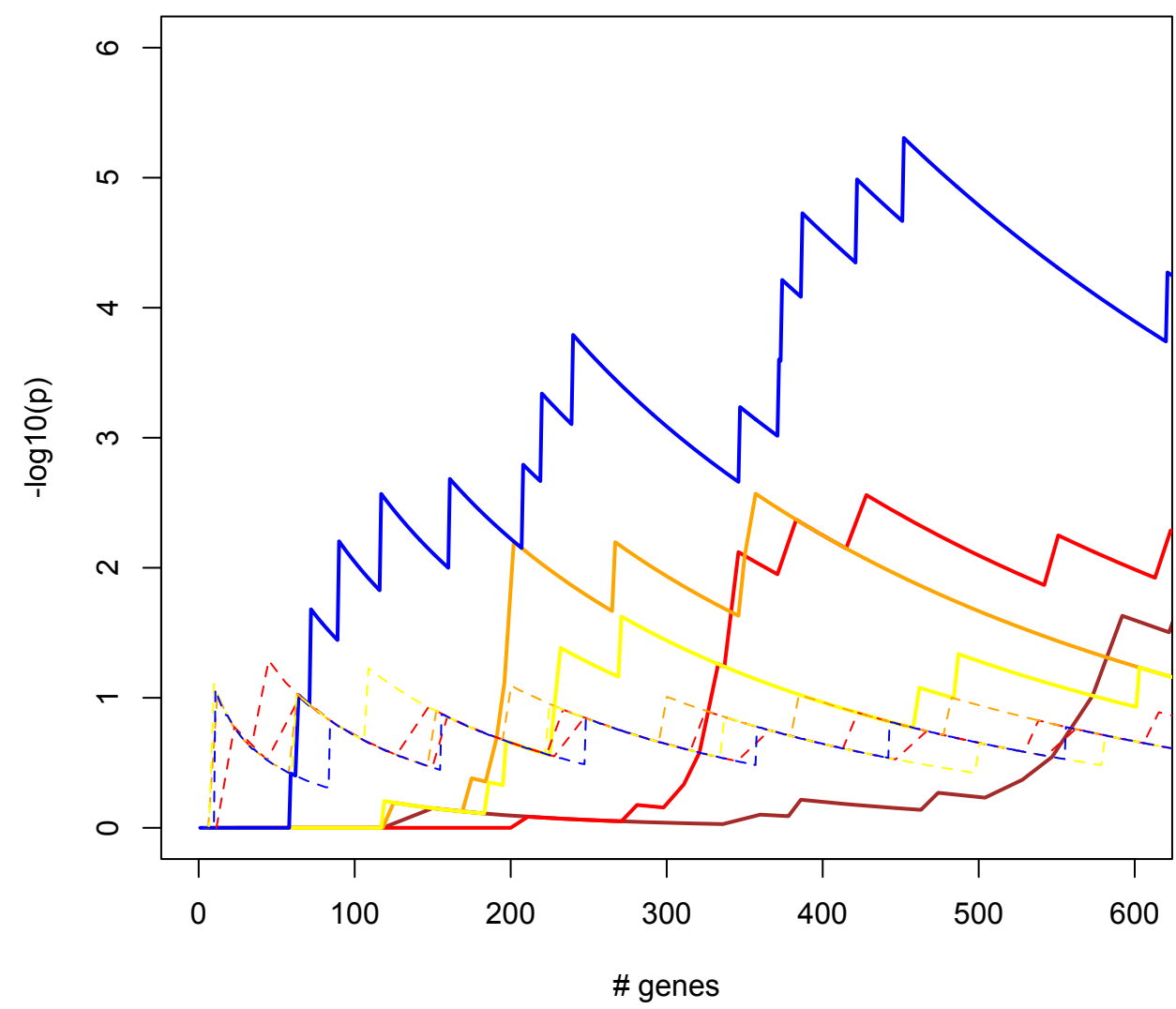

en

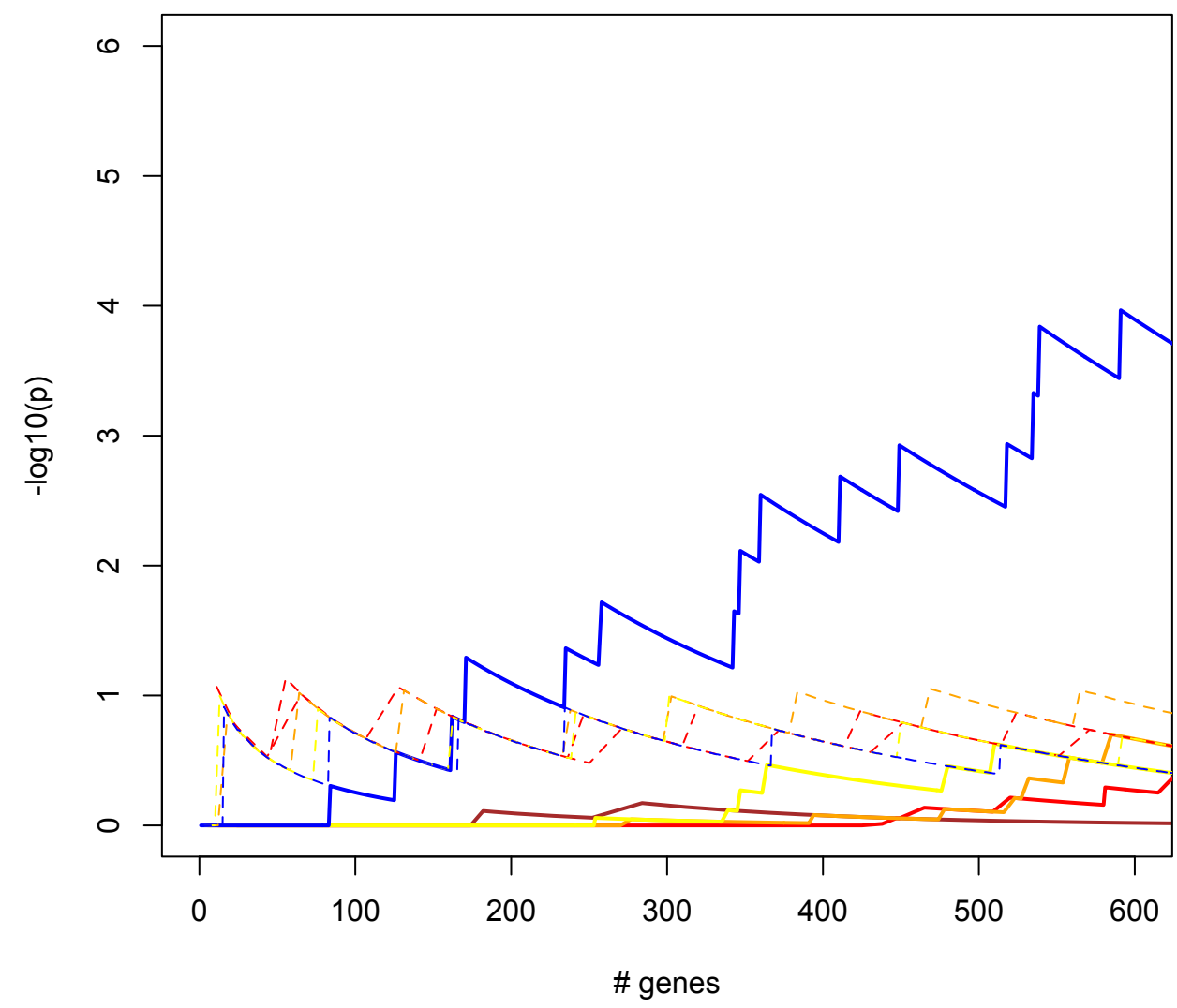

rf

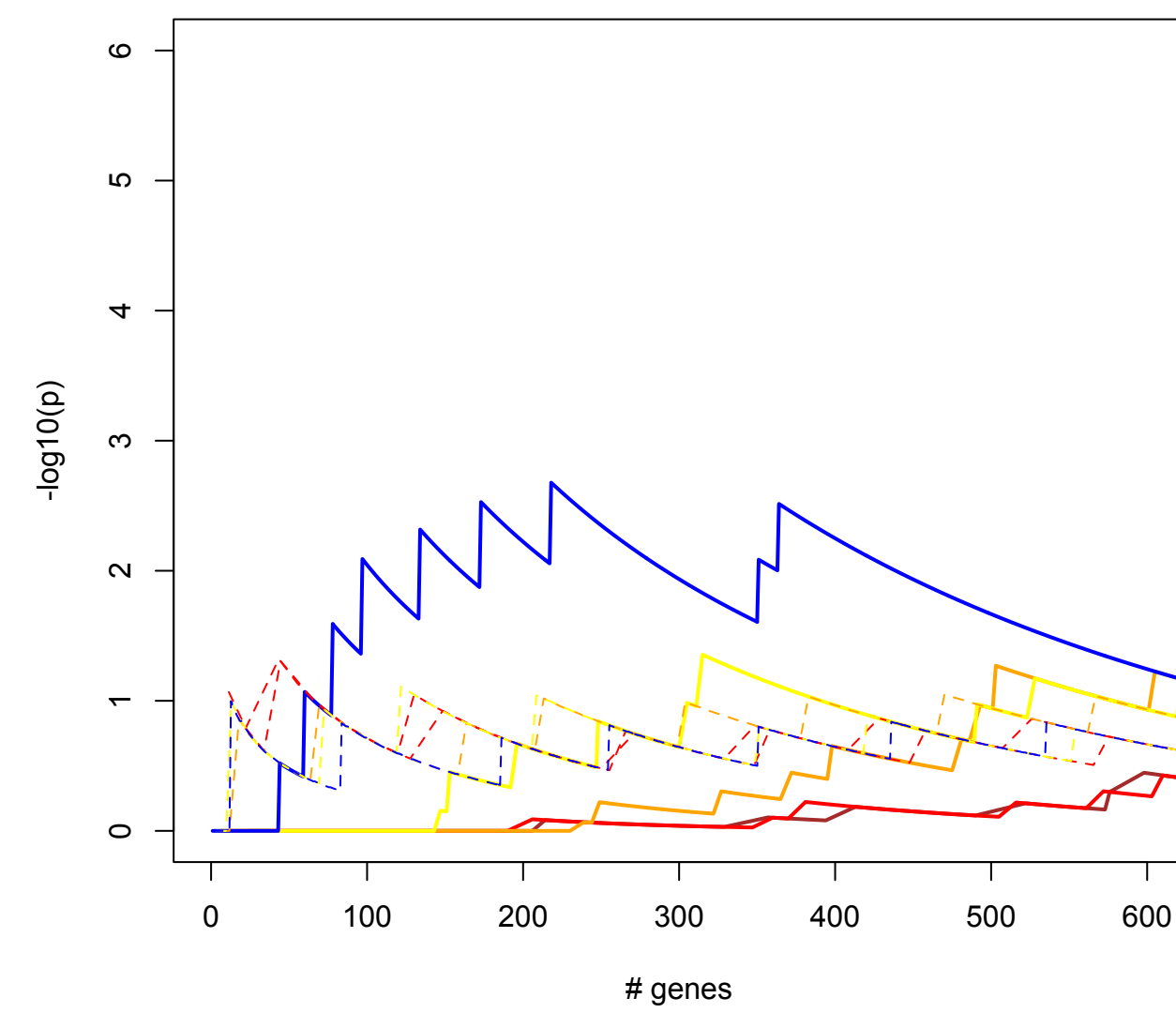

cca

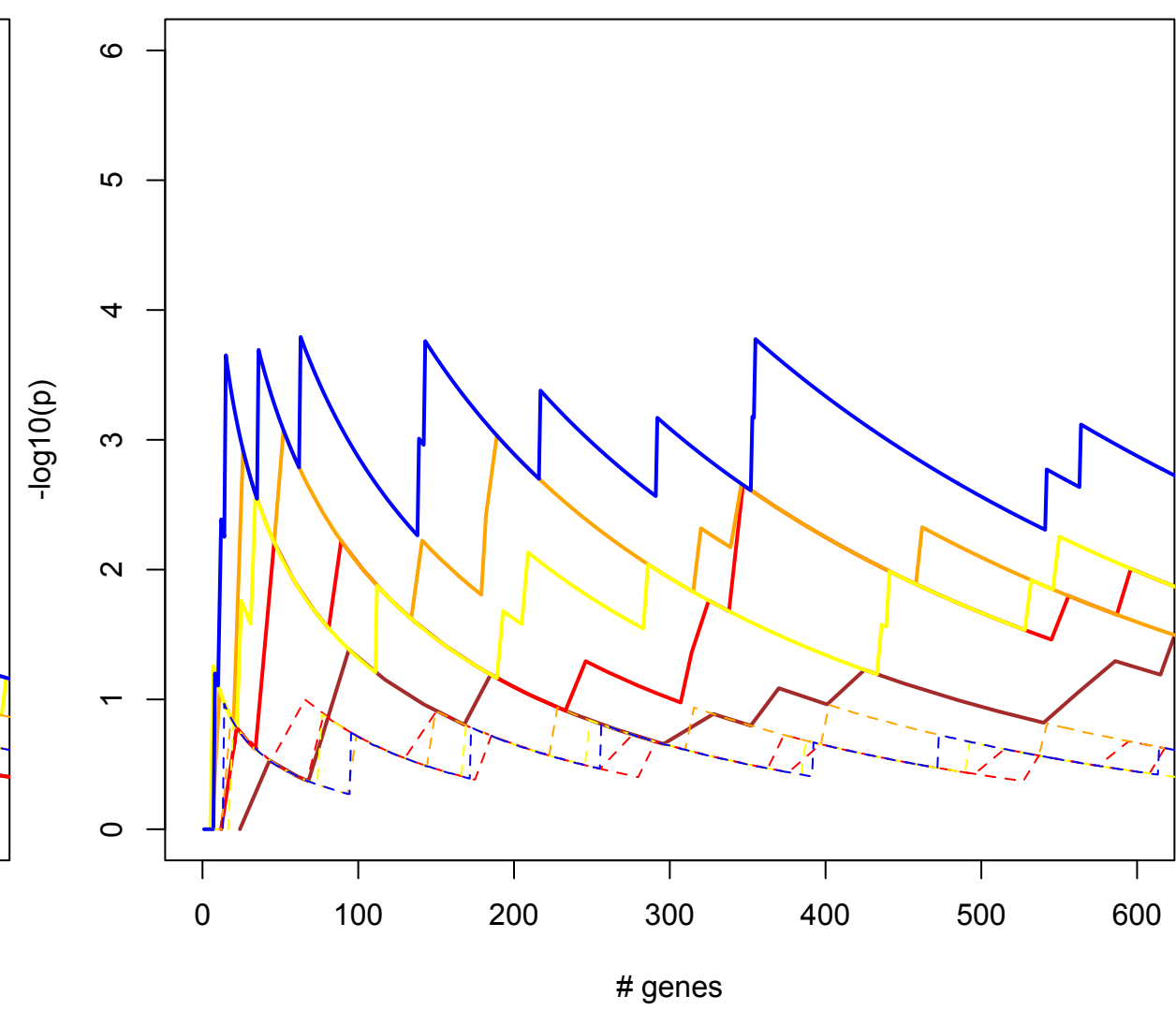

univariate

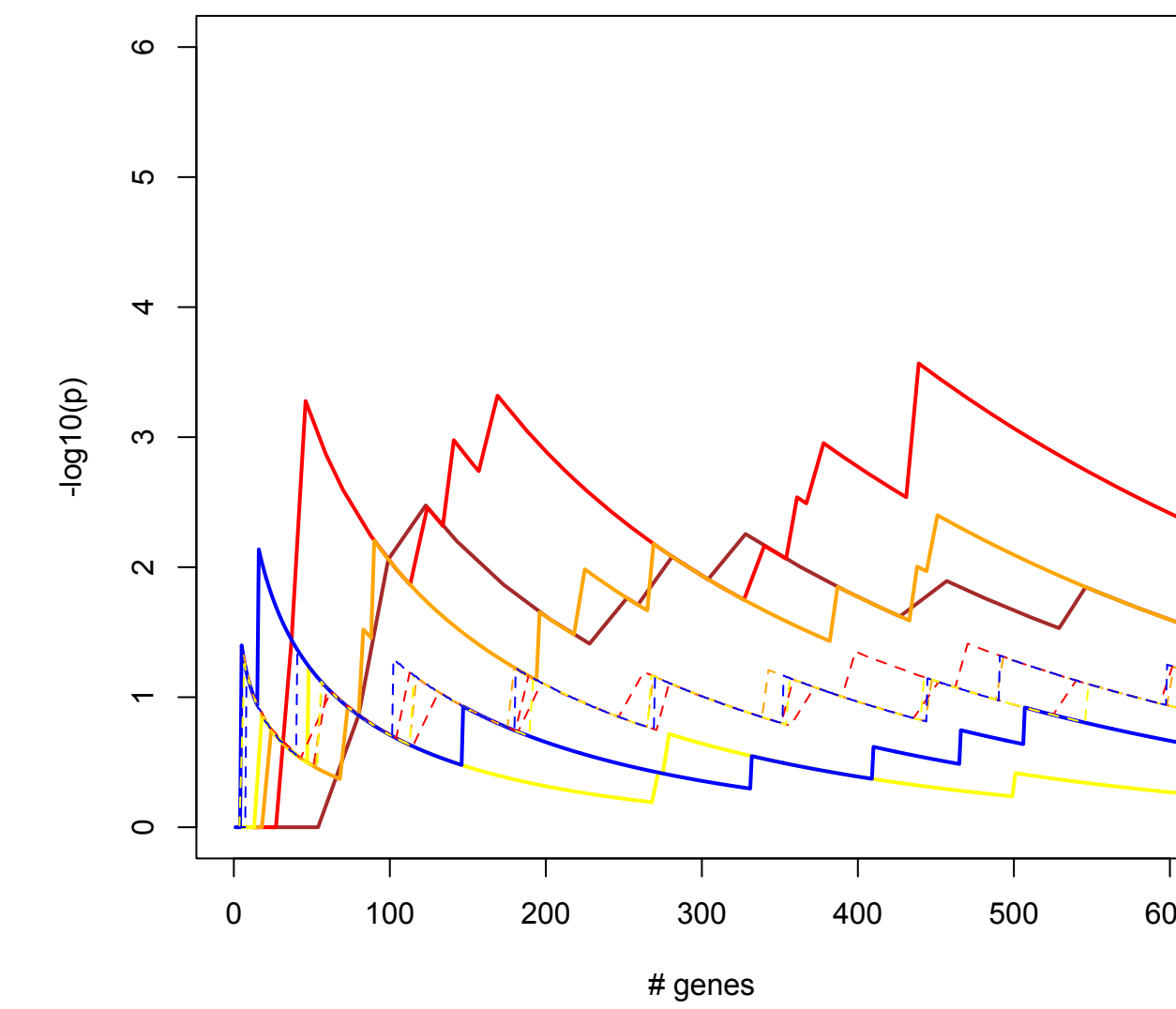

univariate, corrected

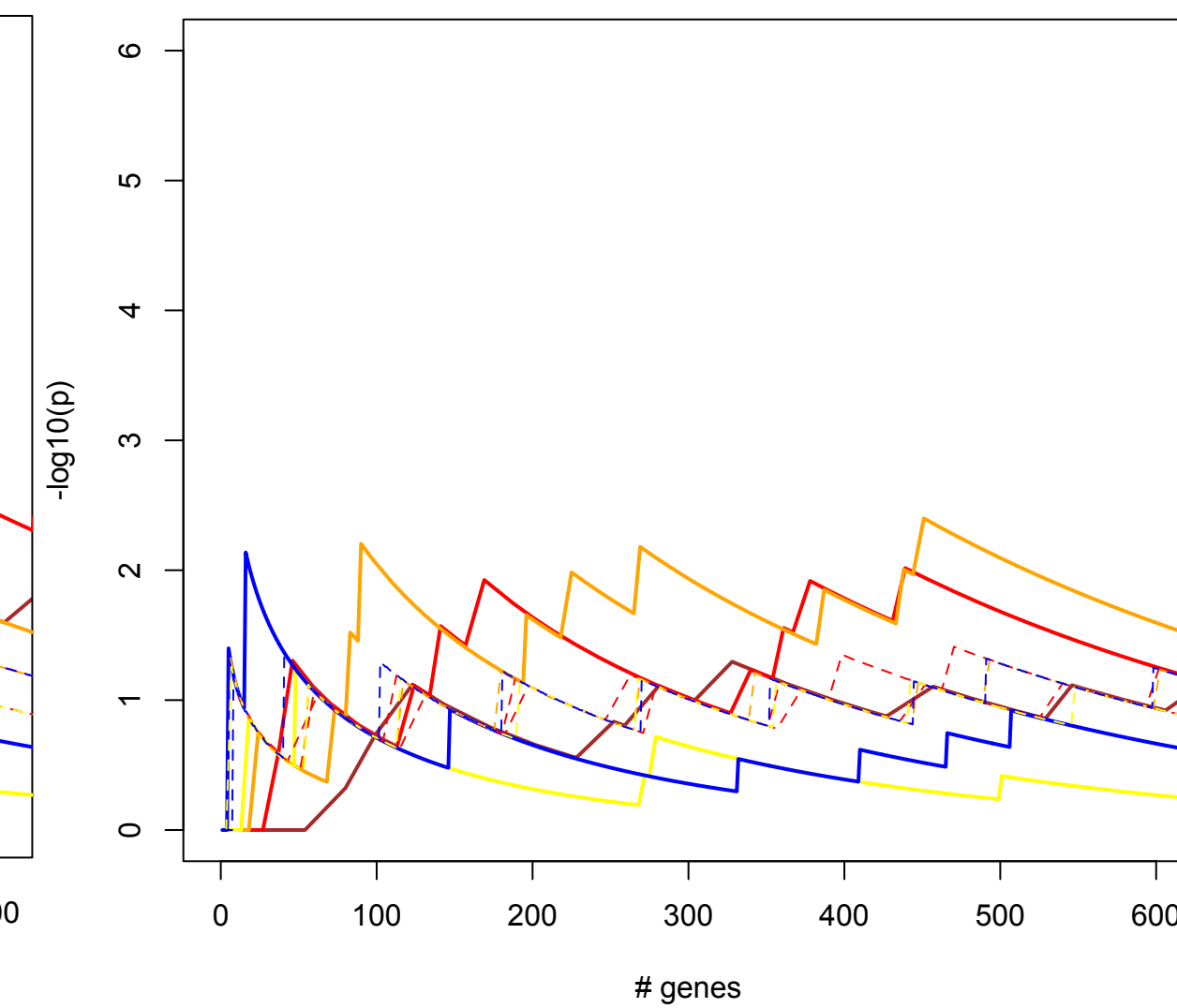

Supplement: S16 Fig — The bottom right panel shows results for univariate mapping of the observed trait when calling a maximum of one gene per SNP (i.e. correction for “double hits”). (PDF) [file pgen.1005594.s016.pdf]
