# Supplementary material for: Genome-Wide Association Analysis of Adaptation Using Environmentally Predicted Traits
Source: PLoS Genet. 2015 Oct 23;11(10):e1005594. doi: 10.1371/journal.pgen.1005594 (PMC4619680; doi:10.1371/journal.pgen.1005594)
Supplement: S14 Fig — (PDF) [file pgen.1005594.s014.pdf]

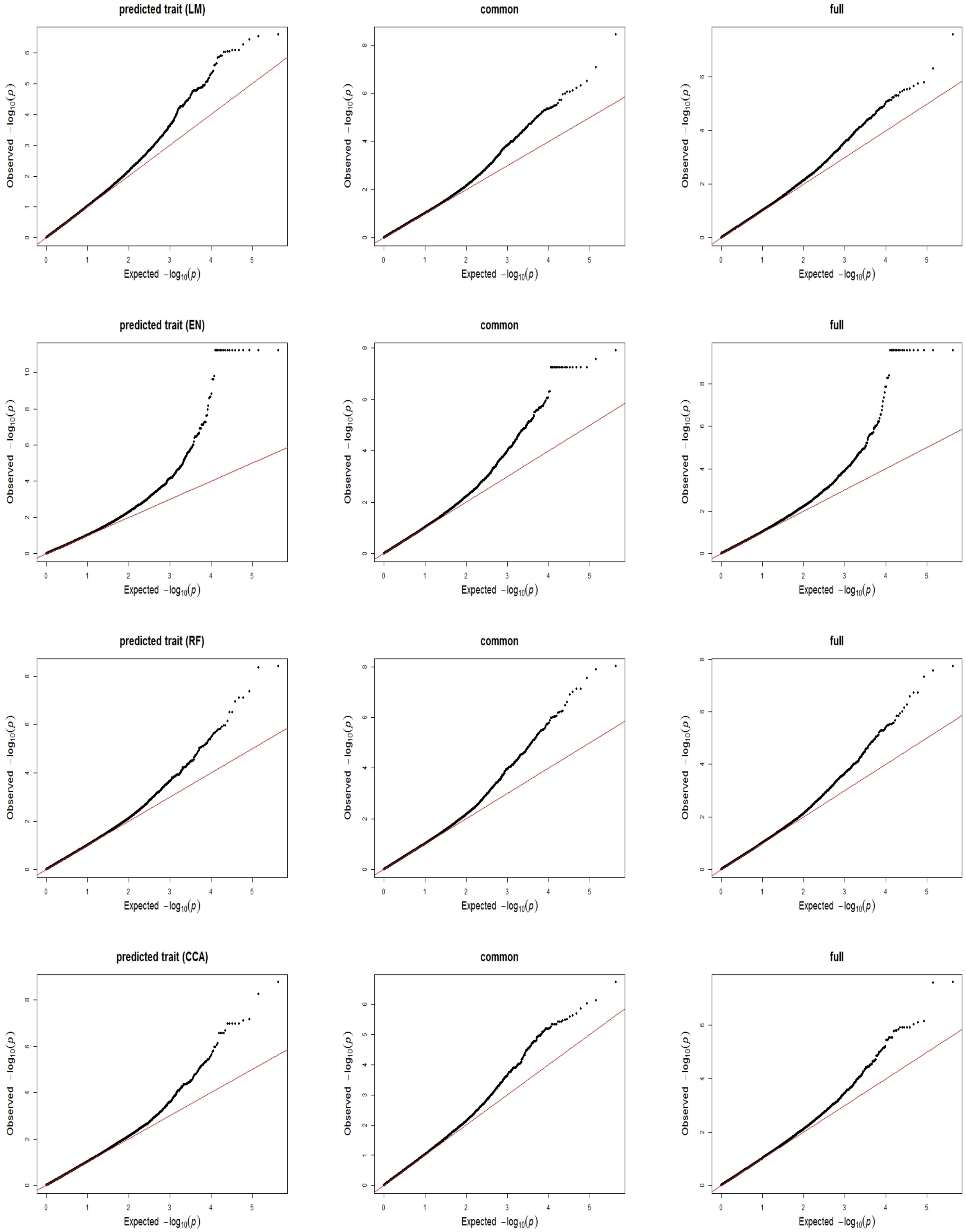
predicted trait (LM)
common
full
predicted trait (EN)
common
full
predicted trait (RF)
common
full
predicted trait (CCA)
common
full
Observed $-\log_{10}(p)$
Expected $-\log_{10}(p)$
